# Dissecting cell diversity and connectivity in skeletal muscle for myogenesis

**DOI:** 10.1038/s41419-019-1647-5

**Published:** 2019-06-03

**Authors:** Yi-xiao Liu, Bing-bing Wu, Lin Gong, Cheng-rui An, Jun-xin Lin, Qi-kai Li, De-ming Jiang, Kai-xiu Jin, Asma Mechakra, Varitsara Bunpetch, Yu Li, Yi-wei Zou, Hong-Wei Ouyang, Xiao-Hui Zou

**Affiliations:** 1 Clinical Research Center, the First Affiliated Hospital, School of Medicine, Zhejiang University, Hangzhou, Zhejiang P. R. China; 2 Dr. Li Dak Sum & Yip Yio Chin Center for Stem Cell and Regeneration Medicine, Zhejiang University, Hangzhou, Zhejiang 310003 P. R. China; 3 Zhejiang Provincial Key Laboratory of Tissue Engineering and Regenerative Medicine, Hangzhou, Zhejiang 310058 P. R. China; 4 College of Agriculture & Biotechnology, Zhejiang University, Hangzhou, 310058 P. R. China; 5 School of Mathematical Sciences, Zhejiang University, Hangzhou, 310058 P. R. China; 6 The Zhejiang University-University of Edinburgh Institute, Zhejiang University School of Medicine, Hangzhou, 310058 P. R. China

**Keywords:** Extracellular signalling molecules, Muscle stem cells, Muscle stem cells

## Abstract

Characterized by their slow adhering property, skeletal muscle myogenic progenitor cells (MPCs) have been widely utilized in skeletal muscle tissue engineering for muscle regeneration, but with limited efficacy. Skeletal muscle regeneration is regulated by various cell types, including a large number of rapidly adhering cells (RACs) where their functions and mechanisms are still unclear. In this study, we explored the function of RACs by co-culturing them with MPCs in a biomimetic skeletal muscle organoid system. Results showed that RACs promoted the myogenic potential of MPCs in the organoid. Single-cell RNA-Seq was also performed, classifying RACs into 7 cell subtypes, including one newly described cell subtype: teno-muscular cells (TMCs). Connectivity map of RACs and MPCs subpopulations revealed potential growth factors (VEGFA and HBEGF) and extracellular matrix (ECM) proteins involvement in the promotion of myogenesis of MPCs during muscle organoid formation. Finally, trans-well experiments and small molecular inhibitors blocking experiments confirmed the role of RACs in the promotion of myogenic differentiation of MPCs. The RACs reported here revealed complex cell diversity and connectivity with MPCs in the biomimetic skeletal muscle organoid system, which not only offers an attractive alternative for disease modeling and in vitro drug screening but also provides clues for in vivo muscle regeneration.

## Introduction

Skeletal muscles have a primary function of locomotion and are responsible for the structure and regulation of the body. Since skeletal muscles are prone to constant injuries due to weight-bearing exercises and traumas, they exhibit a strong regenerative potential. Muscle stem cells, also called satellite cells (SCs), were identified as the key player for muscle repair and regeneration process. These specific cells, which are found juxtaposed to myofibres beneath the basal lamina, are marked by the expression of Pax7^1^. In response to injuries, SCs are activated, undergo self-renewal, and fuse to damaged myofibers to form new ones^1–3^.

For muscle regeneration, the slow-adhering skeletal muscle myogenic progenitor cells (MPCs), or activated SCs, have been widely used in research for skeletal muscle tissue engineering^4^. However, skeletal muscle regeneration has been shown to be coordinated and regulated by various cell types. Previous studies showed the participation of skeletal muscle interstitial cells were found to be a critical regulator of muscle fiber development and maturation^5–7^ or exhibit in vitro and in vivo myogenic capability^8^, such as FAPs (fibro/adipogenic progenitors)^7^ and TCF4^+^ (transcription factor 4) cells^5,6^. However, without clear functions, a group of skeletal muscle-derived rapidly adhering cells (RACs) was identified and described as fibroblast-like cells,y abandoned for their lack of myogenic capability.

The traditional 2D (two-dimensional) MPCs culturing system for skeletal muscle regeneration is limited by a short lifespan and its inability to imitate cell–cell and cell–matrix interactions^9,10^. To overcome these challenges, 3D (three-dimensional) culture systems emerged as an alternative pathway for skeletal muscle tissue engineering^11,12^. Given the heterogeneous cellular nature of the skeletal muscle, the development of a biomimetic in vitro 3D model would be of great value for skeletal muscle physiopathology studies. The organoid system leverages the self-organizing properties of mammalian pluripotent cells or adult stem cells to create 3D in-vitro-grown multicellular clusters with near-native micro anatomy^13,14^. A plethora of organoids has been generated in recent years and multiple reports, acknowledging the capability of this system to recapitulate the body’s multi-level complexity^15–17^, which further allows for the improvement of developmental models for regeneration and pathological studies in complex tissues.

In this study, we explored the function of RACs by co-culturing them with MPCs in a 3D organoid system. We found that RACs significantly promoted the myogenesis of MPCs in the reconstructed organoid. Using single-cell RNA-Seq technology, RACs have been classified into 7 subpopulations, one of which have not previously been reported. In silico ligand–receptor calculating and trans-well experiments showed that growth factors (heparin-binding EGF like growth factor, HBEGF; vascular endothelial growth factor A, VEGFA) and extracellular matrix (ECM) proteins secreted by RACs are key players in the process of skeletal muscle organoid formation.

## Results

### RACs promote myogenesis capability of MPCs in skeletal muscle organoid culturing system

**Fig. 1:**
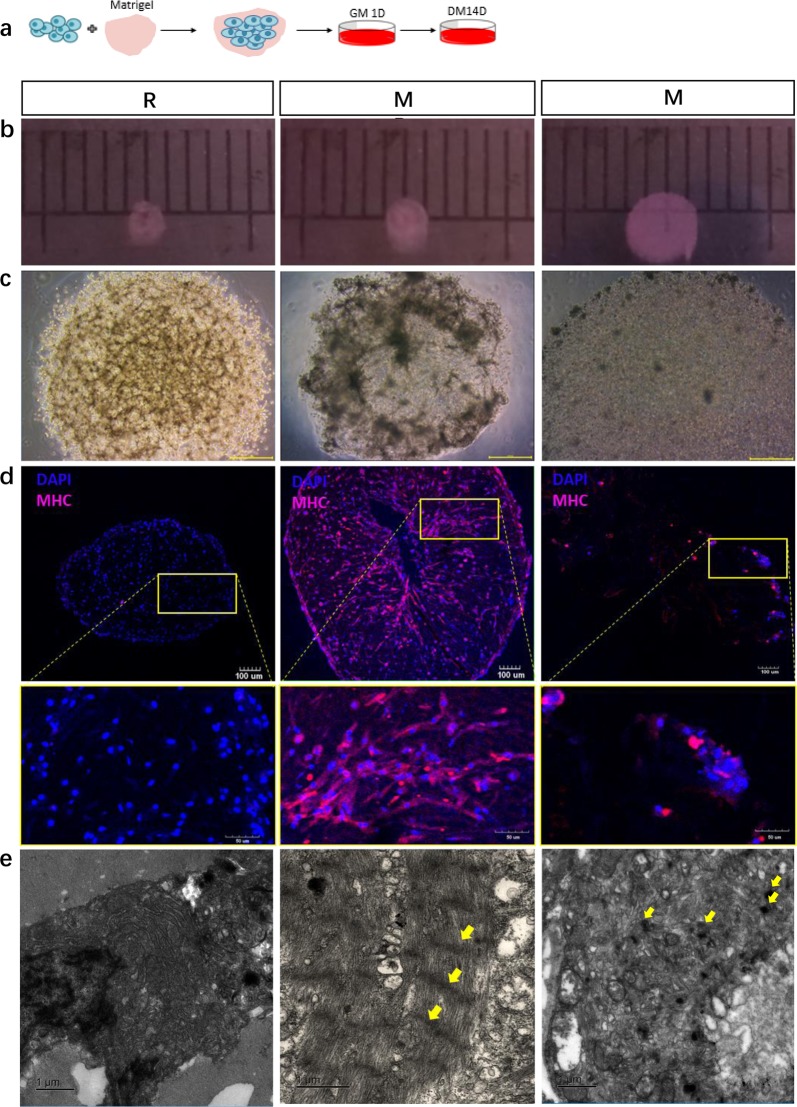
RACs promote myogenic capability of MPCs. **a** Workflow of the protocol to generate skeletal muscle organoid from seed cells and matrigel via myogenic cultured in differentiation medium (DM). **b** Images of skeletal organoid under camera during formation after 14 days culture, R: RACs as seed cells cultured alone, MR: MPCs and RACs co-cultured, M: MPCs as seed cells cultured alone, Scale bars, 1 mm. **c** Images of skeletal organoid under ×40 microscope during formation after 14 days culture, Scale bars, 100 μm. **d** Representative images of myosin heavy chain (MHC) immunofluorescence staining in skeletal muscle organoid three groups: R, MR, M. **e** Ultrastructure of skeletal muscle organoid at 2 weeks of culturing. From left to right: Transmitted electron microscopy images of MPCs and RACs co-cultured in 3D culturing environment for 2 weeks (yellow arrowhead: z lines); Transmitted electron microscopy images of RACs cultured in 3D culturing environment for 2 weeks; Transmitted electron microscopy images of MPCs cultured in 3D culturing environment for 2 weeks (yellow arrowhead: z lines)

Using the preplate technique, we isolated the RACs in addition to the slowly adhering cells (SACs, mainly composed of MPCs) from mice skeletal muscles^4,18^ (Fig. S1a, b). After two purification processes, we obtained purified MPCs where myogenic cells constituted more than 83% of the cell population (Fig. S1c, d). The skeletal muscle organoid was subsequently constructed through the self-organization process of RACs and MPCs in the 3D matrigel in vitro. In early phase (0~24 h), the mixture of RACs and MPCs aggregated rapidly, resulting in a 50% diameter reduction when compared with the monoculture of MPCs, thus leading to a formation of a cohesive multicellular unit (Fig. S2a, b). After the first 24 h culture in growth medium (GM), skeletal muscle organoids were transferred to differentiation medium (DM) and the cells were maintained in culture for an addition of 14 days (Fig. 1a). We then performed immunofluorescence staining of myosin heavy chain (MHC) to assess the myogenic potential of various groups. Compared to the monoculture group, the co-cultured group showed superior myogenic potential (Fig. 1b–d). In addition, from transmission electron micrographs, the 2-week co-cultured group conformed classic sarcomeres (Fig. 1e, MR), while the MPCs monoculture group conformed sarcomeres with malformed Z line (Fig. 1e, M) and there were no sarcomeres in RACs monoculture group (Fig. 1e, R). under a 40 × bright objective, the co-culture mixture of RACs and MPCs was significantly more contractile (supplementary movie M, movie R, movie RM). These results suggested that RACs could enhance the myogenic potential of MPCs in our skeletal muscle organoid systems.

### Single-cell RNA profiling reveals the cell composition of RACs

**Fig. 2:**
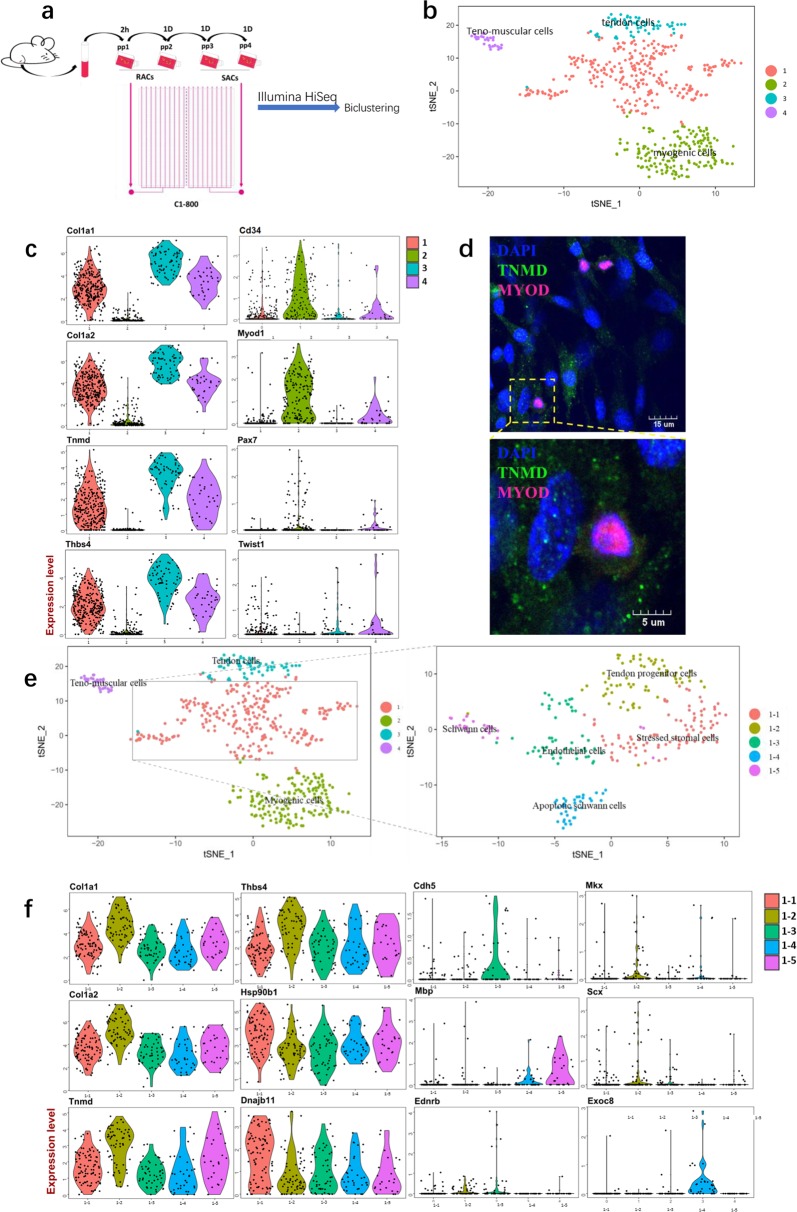
Single-cell RNA-Seq uncovers RACs heterogeneity. **a** Workflow showing the collection of RACs and SACs by preplate technique and RACs and SACs. separately loaded on C1 high-throughput IFC for single-cell cDNA libraries, then by illumine HiSeq platform acquire single-cell RNA-seq library. **b** t-distributed stochastic neighbor embedding (t-SNE) plot of RACs and SACs reveals 4 cell clusters. **c** Violin plots showing the gene expression characteristics of cluster1, cluster2, cluster3, and cluster4 from mice skeletal muscle. **d** Representative images of tendon cells specific marker (Tnmd) and myo-tendon cells, a new cell type not reported before, markers (Myod1, Tnmd) immunofluorescence staining. **e** t-SNE plot of cluster1 reveal five subclusters: cluster1–1, cluster1–2, cluster1–3, cluster1–4 and cluster1–5. **f** Violin plots showing the gene expression characteristics of the 5 sub-clusters

According to the isolation method described by Burhan G. and colleagues^4^, RACs are considered fibroblast-like cells as a whole. To our best knowledge, there are no reports describing RACs is a heterogeneous or homogeneous population or any general studies describing the mammal skeletal muscle cell atlas. Thus, we sought to determine the cellular identity and transcriptomic profile of RACs using single-cell RNA-seq (Fig. 2a).

The t-distributed stochastic neighbor embedding (t-SNE) bioinformatic analysis of transcriptome sequencing data from mice skeletal muscles resulted in 4 clusters: cluster1, cluster2, cluster3, and cluster4 (Fig. 2b). Cluster1, cluster3, and cluster4 are mainly included RACs, while cluster2 was composed of MPCs (Fig. S3a, b). The gene set enrichment analysis (GSEA) revealed ECM protein expression and ECM organization as the expression features of RACs, whereas the expression characteristics of MPCs were myogenic and muscle development (Fig. S3c, d). Based on the expression patterns of cluster-specific genes, cluster2 was *Myod1*^*+*^ and thus classified as myogenic progenitor cells^19,20^. Cluster3 has been classified as tendon cells, specifically expressing *Col1a1, Col1a2, Tnmd*, and *Thbs4*^21^ (Fig. 2a, b). Cluster4 shared myogenic progenitor cell (*Pax7*, *Myod1*)^20,22^ and tenogenic (*Tnmd, Thbs4*) markers, and were subsequently named teno-muscular cells (TMCs) (Fig. 2a, b).

Cluster1 is a more complex group where a detailed analysis identified five sub-clusters within the cluster (Fig. 2e, f). Genes of the heat shock protein family *Hsp90b1* and *Dnajb11 (Hsp40)* were specifically expressed in cluster1–1 which with chaperone-mediated protein folding, ubiquitin-dependent ERAD pathway and endoplasmic reticulum unfolded protein response GO (gene ontology) characteristics (Fig. S5a). Simultaneously, this sub-cluster also specifically expressed the stromal cell characteristic makers *Mmp3* and *Sdf2l1*. On this basis, cluster1-1 has been classified as stressed stromal cells. In addition to tendon cell markers, cluster1–2 also expressed tendon progenitor cells’ markers *Mkx* and *Scx*^23,24^. Combination of the tendon development and collagen fibril organization GO features (Fig. S5b), cluster1–2 has, therefore, been classified as tendon progenitor cells. Cells in cluster1–3 have been classified as endothelial cells given the specific expression of VE Cadherin (*Cdh5*) (Fig. 2f) and epithelium development GO characteristics (Fig. S5c). Cluster1–4 and cluster1–5 specifically expressed *Mbp*. But different with cluster1–5, cluster1–4 specifically expressed *Exoc8* and with apoptotic GO results (Fig. S5d). So we named cluster1–4 as apoptotic Schwann cells and cluster1–5 as Schwann cells^25^.

Taken together, our data suggested the existence of 7 cell subtypes composing the RACs and one cell type in SACs. Tendon cells and tendon progenitor cells were shown to be derived from the connective tissues between myotubes^26^. MPCs^27^, stromal cells^28^, endothelial cells^29^, and Schwann cells^30,31^ have been reported in the past skeletal muscle research. MPCs played a key role in skeletal muscle regeneration^27^. Stromal cells, endothelial cells, and Schwann cells are played collaboration role in skeletal muscle development, homeostasis, and regeneration^5,28,31,32^. However, TMCs was a new cell type not reported before.

### Connectivity map predicts interactions between RACs and MPCs

**Fig. 3:**
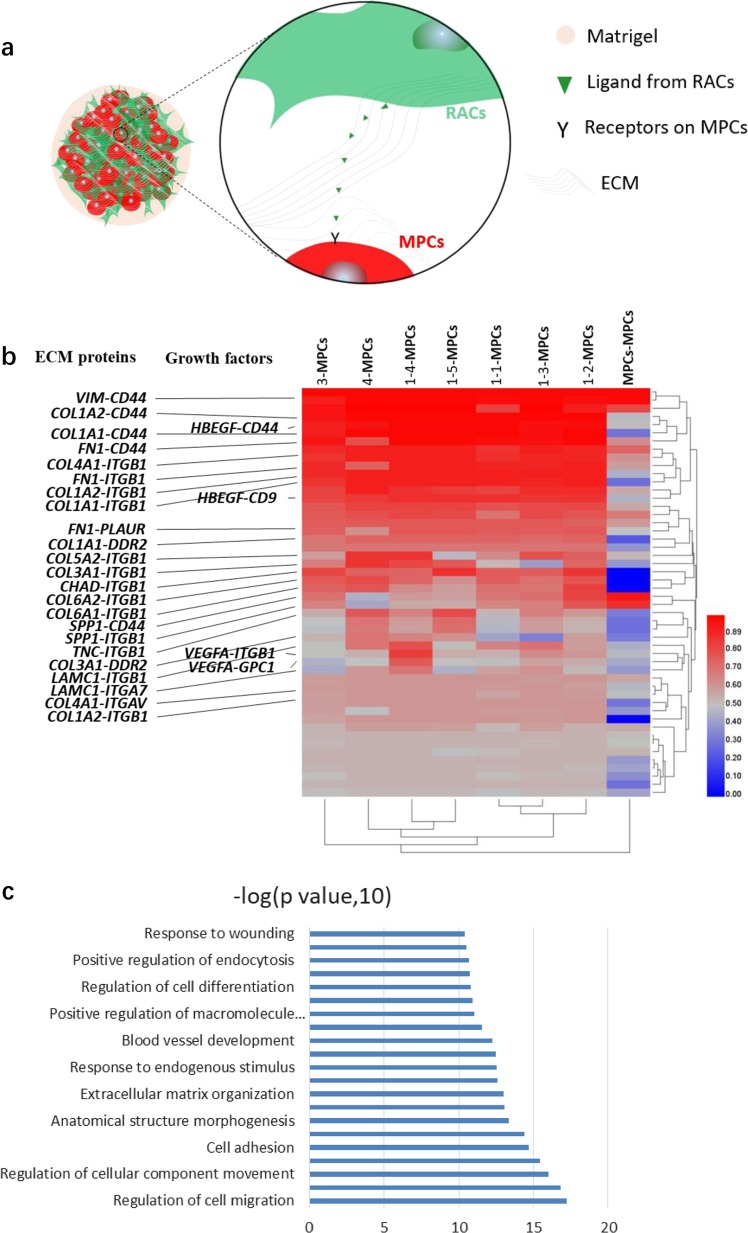
Connectivity map reveals ECM and paracrine signals promote muscle organoid formation. **a** Schematic showing receptor–ligand pairing screen between RACs and MPCs with examples of paracrine. **b** Heatmap showing the mean number of cell–cell interactions per cell type of RACs with MPCs for selected receptor–ligand pairings. **c** GO of the top 50 receptor–ligand parings that participate the cell–cell interaction of RACs with MPCs

We aimed then to determine how the co-cultured RACs increased MPCs myogenic efficiency. We hypothesized that both ECM and growth factors secreted by RACs and cell–cell interactions may play a positive role in MPC proliferation and/or differentiation in the process of skeletal muscle formation (Fig. 3a).

We used in silico receptor–ligand pairing screen method^33^ to identify potential signaling mechanisms underlying the responses observed in 3D skeletal muscle organoids experiments. We calculated the number of potential interactions between RACs and MPCs by determining the presence of a complementary receptor or ligand and summarized potential interaction in the heatmap (Fig. 3b). We found that ECM proteins VIM, FN1, COL1, COL3 COL4, COL5, and COL6, specifically secreted by the 7 RACs’ subpopulations, have the most potential interactions with myogenic MPCs (Fig. 3b). At the same time, GO for top 50 ligand–receptor interactions showed an enrichment in extracellular matrix organization, cell adhesion, cell differentiation, cell migration, and blood vessel development (Fig. 3c). Thus, the result suggested that ECM proteins play an important role in regulating MPC proliferation and differentiation processes. We also found that RACs secreted two growth factors, HBEGF and VEGFA, mediated hot cross-talk with MPCs (Fig. 3b). In our single-cell RNA-seq data, both HBEGF and VEGFA were secreted by all seven subpopulations of RACs. HBEGF was secreted homogeneously, whereas VEGFA was specifically highly expressed in sub-clusters 1–3 and 1–4. The screening results predicted that ECM proteins, HBEGF and VEGFA, mediated the interactions between RACs and MPCs.

### RACs secretome is indispensable during skeletal muscle organoid formation

**Fig. 4:**
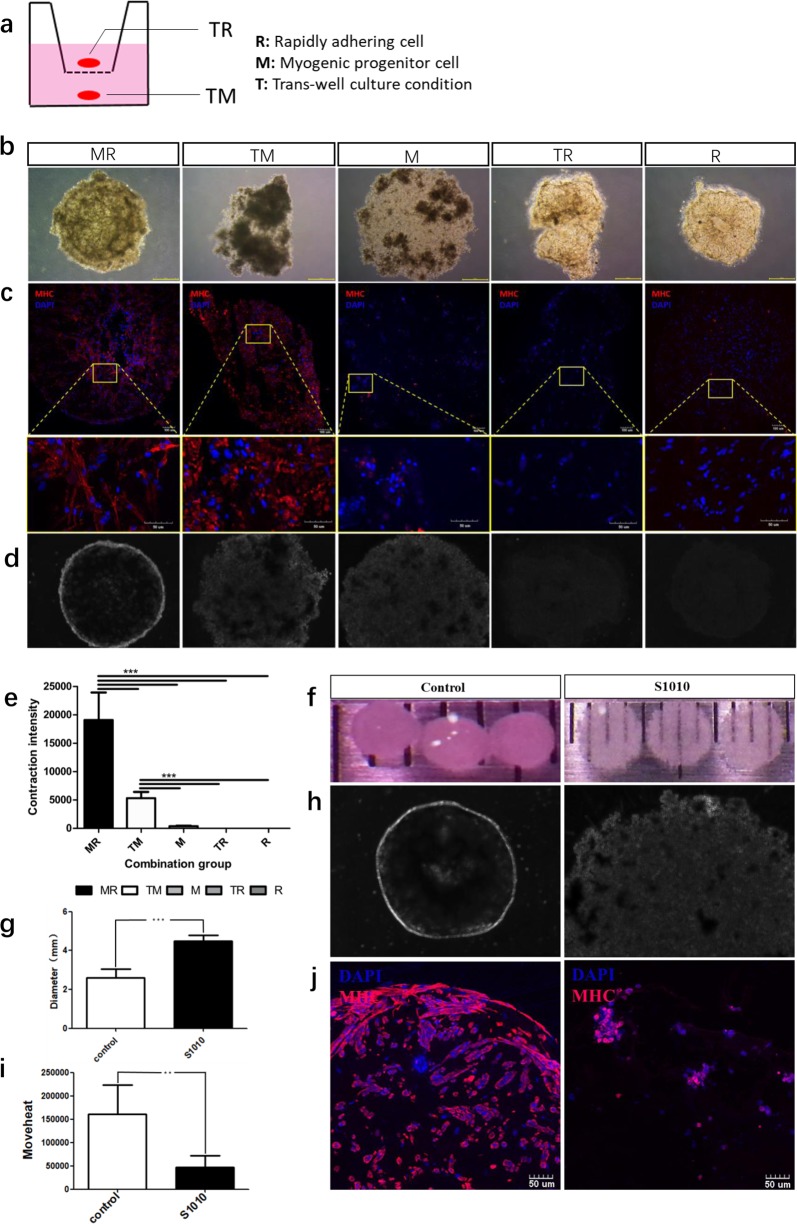
RACs secretome is indispensable during skeletal muscle organoid formation. **a**. Overview of the trans-well culture system, in which RACs and MPCs are cultured separately in a same well but can’t contact with each other. **b** Images of five groups of skeletal muscle organoids under ×40 microscope during formation after 14 days: MR (MPCs and RACs co-culture), TM (Trans-well cultured MPCs), TR (Trans-well cultured RACs), M (MPCs cultured alone), R (RACs cultured alone). **c** Representative images of myosin heavy chain (MHC) immunofluorescence staining slices of skeletal muscle organoid from five groups, Scale bar, 100 μm. **d** Figure generated after contractive intensity quantified by Moveheat (Methods) according to contraction video, the brighter in the figure the stronger of organoid contraction intensity. **e** Quantitative statistics of contraction intensity of MR, TM, TR, M, R (*n* = 3, **p < 0.01, ***p < 0.001). **f** Images of s1010 treated skeletal muscle organoid group and control group. **g** Diameter divergence statistic results of s1010 treated skeletal muscle organoid and vehicle control group. **h** Figure generated after contractive intensity quantified by Moveheat (Methods) according to contraction video of s1010 treated skeletal muscle organoid and vehicle control group. The brighter in the figure the stranger of organoid contraction intensity. **i** Quantitative statistics of contraction intensity of s1010 treated skeletal muscle organoid and vehicle control group. (*n* = 3, *p < 0.05). **j** Representative images of myosin heavy chain (MHC) immunofluorescence staining of s1010 treated skeletal muscle organoid and vehicle control group

According to in silico receptor–ligand pairing screen analysis predictions, RACs derived ECM proteins and growth factors showed close relationship with MPC. We utilized the trans-well strategy to further validate the functions of the ECM proteins and growth factors in the process of skeletal muscle organoid formation (Fig. 4a). First, MHC immunofluorescence staining of the 14-day trans-well culture organoid (Fig. 4b) showed a higher myogenic differentiation efficiency in both the co-culture group and trans-well group (RACs and MPCs) when compared to the MPCs monoculture group (Fig. 4c). In order to quantify the contraction intensity of the skeletal muscle organoid, we developed the Moveheat calculation method (described in the “Methods” section). The Moveheat statistical results (Fig. 4d, e) showed a significantly higher contraction function in the co-culture group in comparison to the other groups. Interestingly, no significant differences in the MHC immunofluorescence staining were detected between the co-culture and trans-well group. However, the contraction intensity was weaker in the trans-well group suggesting that RACs-secreted growth factors could be transferred to MPCs. This confirmed that some of the ECM proteins and growth factors from RACs could penetrate though the trans-well and promoted the myogenesis of MPCs. According to the connectivity map, RACs derived VEGFA showed high cross-talk with receptors (IGTB1 and GPC1) on MPCs. So, in order to further verify the function of VEGFA in the formation of the skeletal muscle organoid, we utilized small molecular drug inhibitor (Nintedanib, s1010) of VEGFA receptors to block the receptors of VEGFA and found that not only the great divergence of organoid diameters (Fig. 4f, g) between the s1010 treated group and vehicle control group but also the myogenesis of skeletal muscle organoids was inhibited (Fig. 4j) as well as their contraction function (Fig. 4h, i). Thus, these results confirmed the involvement of VEGF signaling during the organoid formation and implied the potential of the biomimetic muscle organoid system in the scale-up high-throughput drug screening application in vitro.

## Discussion

In this study, we illustrated RACs’ roles in promoting MPCs myogenic differentiation in a novel biomimetic skeletal muscle organoid system. We further dissected cell diversity and connectivity of RACs and MPCs in skeletal muscle myogenesis. Beyond its potential applications in basic research as an improved model for skeletal muscle regeneration, our skeletal muscle organoid system, in combination with the Moveheat method (with a quantifiable parameter allowing a quick characterization based on contraction intensity) is suitable for the scale-up of high-throughput drug screening applications given its simple implementation comparing with previously reported skeletal muscle tissue engineering technologies^11,34^.

Currently, the expansion of MPCs and the formation of myotubes in two-dimensional (2D) systems are the most commonly used models in muscle regeneration research. However, these cultures are not only difficult to maintain over long periods of time but also lack native muscle architecture^35,36^. Also, as growing evidence pinpoints the importance of extracellular matrix in muscle stem cell niche regulation, muscle dystrophies, and wasting disorders^10,37–41^, 2D models are inherently limited in their ability to account for cell–cell and cell–matrix interactions. This limitations led to an increase in 3D culture models in skeletal muscle tissue engineering studies^11,12,34^. However, in most cases, only pure myogenic primary cells or myogenic cell lines are used as seed cells. Given the heterogeneous cellular nature of the skeletal muscle, a biomimetic in vitro skeletal muscle regeneration model is essential to study skeletal muscle health and disease. So in this study, combined with the recently reported organoid technology^14,15^, we constructed a skeletal muscle organoid culturing system by co-culturing RACs with MPCs in a 3D environment. In contrast to the 2D culturing system, we found that RACs played a key role in the formation of skeletal muscle organoid with contractile functions, resulting in a mature skeletal muscle more similar to the native tissue.

Moreover, we investigated the RAC populations' composition and functions. Using single-cell RNA-Seq, we classified RACs into 7 different subpopulations, including one new cell subtype: TMCs. Interestingly, TMCs also specifically expressed Twist1 gene. Cells within this cluster are similar to the Twist2-dependent myogenic progenitor cells, which are distinct from SCs reported recently by Ning Liu et al.^42^ TMCs showed a cellular plasticity by concomitantly expressing myogenic progenitor cell markers and tendon cells’ classical phenotypic markers. However, when cultured alone (monoculture groups) in the skeletal muscle organoid system, RACs did not exhibit a myogenic phenotype, suggesting that in vitro environment cannot induce TMCs into myogenic differentiation. In addition, former study reported skeletal muscle-derived interstitial cell types such as TCF4^+^ cells^5^, myogenic endothelia cells^43^, fibro/adipogenic progenitors (FAPs)^7^, PW1^+^/Pax7^−^ interstitial cells (PICs)^8^ and pericytes^41^ could be found in our single-cell RNA-seq data (Fig. S4a, b), but the twist2^+^ cells which reported by Ning Liu et al. could not be found. Probably this because the amount of cells which be analyzed is too small or the depth of single-cell RNA-seq is not enough to find genes with low abundance expression. MPCs myogenesis is promoted by RACs has been previously reported by Sam J. Mathew et al.^5^ and Male M. Murphy et al.^6^ In their study, TCF4+ connective tissue fibroblasts were reported to interact with SCs and to play a key role in skeletal muscle myogenesis. However, our single-cell RNA-seq showed that TCF4 transcripts are not only expressed in all RACs sub-clusters but also expressed in Myod1^+^ cells (Fig. S4a, b), suggesting the non-specificity of TCF4 as a marker of skeletal muscle connective fibroblasts.

ECM and growth factors are important components of MPCs' niche in the skeletal muscle^39,40,44,45^, but how the niche affects MPCs remains poorly understood. The intersection of our single-cell RNA-Seq data with connectivity map results identified potential signaling between RACs and MPCs. RACs-secreted ECM proteins such as VIM, COL1, COL4, FN1, COL6 showed high interaction scores with their corresponding MPC-expressed receptors. The results of former studies partially consolidate our calculation results, such as Uriuolo A. and collaborators found that collagen VI (COL6) is critical for the preservation of SCs' self-renewal and muscle regeneration ability^37^. Moreover, Lukjaneko L. and colleagues showed that the loss of fibronectin (FN1) from aged stem cell niche affects the regenerative capacity of skeletal muscle in mice^46^.

HBEGF which is widely expressed in all 7 clusters of RACs showed high interaction score with MPCs. VEGFA, which is specifically highly expressed in endothelial and apoptotic Schwann cell clusters, also showed higher interaction score with MPCs. In vivo repairing experiment revealed that local application of VEGF may improve restoration of muscle force by reducing connective tissue and increasing the relative amount of muscle fibers^47,48^. These growth factors have also been reported to play an important role in hepatocyte development^33^.

Finally, the in silico receptor–ligand pairing screen results were verified by our skeletal muscle organoid system in addition to trans-well culture experiments. The micro-environment secreted by RACs, together with ECM proteins and growth factors, played an independent role in promoting myogenesis of MPCs in skeletal muscle organoid system. The ligand–receptor score results provided new findings in relation to MPCs transplantation, skeletal muscle tissue engineering, and skeletal muscle regeneration.

In conclusion, we showed that RACs promoted the myogenic capability of MPCs in a novel skeletal muscle organoid system and partially revealed the mechanism of this phenomenon by cell–cell connectivity map and small molecular inhibitor experiments. At the same time, based on the single cell sequencing data we classified the different cell subpopulations in skeletal muscle RACs. This work provides a more adequate seed-cells choice for skeletal muscle tissue engineering and may contribute to solving existing MPCs transplantation issues.

## Methods

### Animals

All experiments were performed on 6–8 weeks old male C57BL/6 mice, which were purchased from the Zhejiang Academy of Medical Science. All animal experiment protocols were approved by the ethical committee of Zhejiang University School of Medicine (ZJU20170783) and were in compliance with institutional guidelines.

### Preparation of single skeletal muscle cell suspension and preplate protocol

Single cell suspension derived from mice skeletal muscle tissues was prepared as described previously^4^. Briefly, muscle tissues were collected from hind limbs (connective tissues were removed), minced very finely with scissors and digested with collagenase XI (C7657-100 mg, sigma) for 60 min. After 5 min centrifugation (930×*g* at 4 °C), the supernatant was removed and a second digestion was performed for 45 min with dispase II (D4693-1G, Sigma). A second centrifugation was performed (930 × *g* at 4 °C for 5 min) and the supernatant was discarded. The pellet was resuspended in 0.1% trypsin-PBS solution and incubated for 15 min at 37 °C. The resulting suspension was centrifuged at 930 × *g* at 4 °C for 5 min then the resulting pellet was resuspended with GM to form single skeletal muscle cell suspension.

Preplate protocol: (1) We plated the single skeletal muscle cell suspension onto a collagen I (356230, Corning) coated 60 mm dish (labeled as PP1). Petri dishes were incubated at 37 °C/5% CO_2_ incubator for 2 h. We transferred nonadherent cells to a new collagen I coated 60 mm dish (labeled as PP2), then we added 3 ml of GM into the plate labeled pp1. (2) We returned the dishes for additional 24 h incubation before again transferring the nonadherent cells to a new dish marked PP3 and 3 ml of GM was added into the plate labeled PP2. (3) We returned the dishes to the incubator and repeated the procedure for an addition of 24 h, then PP4 was handled according to the preplate technique reported previously^4,18^. PP1 and PP2 adhering cells are fibroblast-like cells and PP3 and PP4 adhering cells are mainly composed of activated SCs and quiescent SCs. By repeating the step (1) twice, we can remove the fibroblast-like cells in PP3 and PP4 and acquire purified myogenic progenitor cells (MPCs).

### Single cell reverse transcription, pre-amplification, library preparation and sequencing

Tibial anterior and gastrocnemius muscles were dissociated from male C57BL/6J mice hind limbs (6–8 weeks, *n* = 2). RACs and SACs were isolated from skeletal muscle using the preplate technology^4^. Single cell transcriptome was then obtained according to the fabricant’s protocol (Fluidigm Corporation) “Using the C1 High-Throughput IFC to Generate Single-cell cDNA Libraries for mRNA Sequencing” (Page32–Page50). In this process, we used C1 High-Throughput IFC (PN100 100–9886, Fluidigm Corporation) to capture single cells. In order to exclude the capture site without or with multiple cells, we observed each capture site under the microscope after the cells were loaded on the chip.

Single cell cDNA libraries for mRNA sequencing was generated by C1 High-Throughput IFC. Single-cell RNA-seq of 562 individual cells, including 336 RACs and 226 SACs, was performed. The RNA-seq library was subjected to 150 bp paired-end sequencing on an illumine HiSeq platform (sequenced by Annoroad Gene Tech. (Beijing) Co., Ltd). We obtained an average of about 2 million reads per cell.

### Computational bioinformatics analysis of single-cell data

Single-cell RNA sequence reads were evaluated by FastQC^49^. Bad reads were detected automatically and removed. Bases at 3′ end of the read were further trimmed by NGS QC Toolkit if their Phred quality score were less than 20^50^. The reads were mapped to the mouse genome (mm9) using Bowtie2. The mapped reads for genes were counted via HTseq^51^.

Quality control, clustering and differential expression analysis were performed using a recently published method using Seurat v2.0^52^.

For all 562 sequenced single cells, we quantified the numbers of genes and transcripts in each cell. Only the genes with an expression level greater than 1 that were expressed in at least 3 cells were considered. Single cells with fewer than 1000 genes expressed were filtered out. In total, 488 cells passed the filter standards. The expression levels were normalized by “LogNormalize” that normalizes the gene expression measurements for each cell by total expression, multiplies this by a scale factor (10000 by default), and log-transforms the results.

With default parameters, FindVariableGenes was used to identify variable genes which were used for initial principal component analysis. We then performed a jackstraw analysis to select principal components (PCs) for their *P*-values were below 6.02e−5. tSNE was performed through the RunTSNE function (dim.use = 1:10). To cluster cells, we used FindClusters. Marker genes of each cell cluster were analysed using the Find All Markers function with logfc.threshold parameter set at 0.25.

### Cell culture studies

During the preplate and MPCs expansion culturing process, cells were cultured in growth medium (GM): High glucose Dulbecco’s Modified Eagle’s medium (H-DMEM), 10% heat-inactivated fetal bovine serum (FBS), 10% heat-inactivated horse serum (HS), 0.5% chick embryo extract (CEE; Accurate Chemical Co., cat. no. CE650T-10), 2 mM l-glutamine, and antibiotics (50 U/ml penicillin and 50 mg/ml streptomycin). In the differentiation culture part, skeletal muscle organoids were cultured in differentiation medium (DM) composing of High glucose Dulbecco’s Modified Eagle’s medium (H-DMEM), 2% heat-inactivated horse serum (HS), and antibiotics (50 U/ml penicillin and 50 mg/ml streptomycin).

### Skeletal muscle organoid construction

To acquire enough cells, RACs and MPCs were cultured on collagen I coated 60 mm dish and incubated in a 5% CO_2_ 37 °C incubator.

For the co-culture system, RACs and MPCs were independently dissociated into single cells using 0.05% trypsin and mixed at a 1:1 ratio. The cell mixture was suspended (3 × 10^6^ cells/ml) in cold GM supplemented with 50% Matrigel (354230, 10 ml, Corning® Matrigel® Matrix Growth Factor Reduced (GFR) then solidified as a droplet (20 μl each) on a 60 mm dish in the incubator. That is there are 3 × 10^4^ RACs and 3 × 10^4^ MPCs in one drop of co-culture system. For the monoculture system, there are 3 × 10^4^ RACs in one drop of RACs monoculture system as well as MPCs monoculture system.

24 h after GM was added, we changed GM to DM and continued culturing for 2 weeks.

### Skeletal muscle organoid contraction intensity measurements

The Moveheat method was established as follows:

(1). Mature skeletal muscle organoids were taken out from the cell culture incubator and their contraction was recorded in a video under ×40 bright microscope. (2). Grayscale differences between every 4 frames in the video were calculated. (3). Finally, the average value of the gray changes in each pixel of all the frames of the video was considered as the change heat in the image of the corresponding region.

### Immunofluorescence

A series of frozen 16 μm thick sections were utilized for immunofluorescent staining. The series of sections were fixed with 4%(w/v) paraformaldehyde for 30 min at 4 °C, rinsed three times with PBSCM (add 0.4 ml of 1 M CaCl_2_, and 0.4 ml of 1 M MgCl_2_ into 400 ml of 1 × PBS), and treated with 0.1% saponin (Sigma s-7900) (add 0.4 g saponin per 400 ml PBSCM) to permeabilize cells for 15 min at room temperature (RT).

Next, primary antibody was diluted in FDB (PBSCM supplemented with 5% normal goat serum, 2% fetal bovine serum, 2% bovine serum albumin) then spun at 14K rpm, 4 °C for 10 min. We used 50 μl of the dilution for each slide and incubated them overnight at 4 °C. Slides were then washed 3 times for 2 min in 0.1% saponin with gentle shaking at RT.

Secondary antibody was diluted in FDB and spun at 14K rpm, 4 °C for 10 min. We used 50 μl of diluted antibody in each slide which were then incubated 2 h at RT. Slides were then washed 3 times for 2 min in 0.1% saponin with gentle shaking at RT. Slides were sealed with FluorSave™ Reagent (Millipore, 345789-20 ml).

We used the following primary antibodies: Myosin MF20 (DSHB), PAX7 (DSHB) and rat anti-mouse secondary antibodies (eBioscience). Goat anti-rabbit secondary antibody (Invitrogen) and DAPI (Beyotime Institute of Biotechnology) were used to visualize the respective primary antibodies and the cell nuclei. All procedures were carried out according to the manufacturer's instructions.

### Image analysis

The images of skeletal muscle organoid were taken by inverted microscope (OLYMPUS XDS-1B) or a stereo microscope (OLYMPUS XTZ-D). Fluorescent images were captured using Olympus FV3000 Laser scanning confocal microscope (OLYMPUS IX83-FV3000). Transmitted electron microscopy images were taken by Cryo-electron microscope (Spirit 120 kV).

### Statistical analysis

The one-way Anova test was used to determine statistical significance and was conducted using GraphPad Prism 5.0 software. *p* < 0.05 was considered to be statistically significant. Experiments were repeated at least three times. The number of repeats is given in the figure legends.

## Supplementary information

Supplementary information

Isolated RACs and MPCs

Diameters of organoid after 24 h

Profiling characteristics of RACs and SACs with single cell RNA-seq

The expression of presentative marker of which have been reported before in skeletal muscle derived cell types

The GO characteristics of C1-1,C1-2,C1-3,C1-4

Using the C1 High-Throughput IFC to Generate Single-cell cDNA Libraries for mRNA Sequencing

The contract situation of MPCs cultured in 3D culturing environment for 2 weeks

The contract situation of RACs cultured in 3D culturing environment for 2 weeks

The contract situation of MPCs and RACs co-cultured in 3D culturing environment for 2 weeks

a video of skeletal muscle organoids pictures gathering
