# Supplementary material for: Dissecting cell diversity and connectivity in skeletal muscle for myogenesis
Source: Cell Death Dis. 2019 Jun 3;10(6):427. doi: 10.1038/s41419-019-1647-5 (PMC6546706; doi:10.1038/s41419-019-1647-5)
Supplement: Supplementary file 1 — Supplementary information [file 41419_2019_1647_MOESM1_ESM.docx]

**Supplementary information:**

**Fig. S1**: Isolated RACs and MPCs.

**a.** RACs under 40× microscope, scale bar 500μm. **b.** MPCs under 40× microscope, scale bar 500μm. **c.** Representative images of PAX7, MYOD1 immunofluorescence co-staining in MPCs, scale bar 100 μm. **d.** Statistic results of the myogenic cells’ proportion in MPCs (n=3).

**Fig. S2**: Diameters of organoid after 24 h.

**a.** The diameter of three organoid groups 24 hours after culturing started. R: RACs cultured alone in matrigel; RM: RACs and MPCs co-cultured in matrigel; M: MPCs cultured alone in matrigel. **b.** The diameter of those three groups after 24h (n=3, *: p<0.05).

**Fig. S3:** Profiling cell composition of RACs with single cell RNA-seq.

**a.** The distribution of RACs (PP1, PP2) and SACs (PP3, PP4) in t-SNE results: RACs are mainly constructed by cluster1, cluster3 and cluster4, while cluster2 is mainly included in SACs. (blue, negative; red, positive) **b.** Statistic results of the percentage of C1, C2, C3, C4 in RACs and SACs. **c.** Expression of known ECM marker genes and myogenic marker genes are shown on t-SNE plots; a gradient of blue and red indicates low to high expression. **d.** The gene set enrichment analysis (GSEA) revealed the expression characteristics of the 4 cluster cells: cluster1, cluster3 and cluster4 specifically expressed genes related with extracellular matrix synthesis, while cluster2 expressed genes related with myogenic and muscle development.

**Fig. S4**: The expression of presentative marker of which have been reported before in skeletal muscle derived cell types.

**a**. Violin plots showing the expression of specific marker genes, which have been reported in former study, in cluster1, cluster2, cluster3 and cluster4. Tcf4 is the marker of Tcf4^+^ skeletal muscle connective tissue fibroblasts, Cdh5 and Ncam1(Cd56) are the marker of endothelial cells, Cspg4 (NG2) and Mcam (Cd146) are the maker of pericytes, Peg3 (Pw1) is the maker of Pw1^+^ cells, Cd34 and Atxn1 (Sca1) are the markers of FAPs. **b.** Violin plots showing the expression of specific marker genes, which have been reported in former study, in cluster1-1, cluster1-2, cluster1-3, cluster1-4 and cluster1-5.

**Fig. S5**: The GO characteristics of C1-1, C1-2, C1-3, C1-4.

**a.** The GO characteristics of C1-1. **b.** The GO characteristics of C1-2. **c.** The GO characteristics of C1-3. **d.** The GO characteristics of C1-4.

**Supplementary movie M**: The contract situation of MPCs cultured in 3D culturing environment for 2 weeks.

**Supplementary movie R**: The contract situation of RACs cultured in 3D culturing environment for 2 weeks.

**Supplementary movie RM**: The contract situation of MPCs and RACs co-cultured in 3D culturing environment for 2 weeks.
