# Supplementary figures and images for: Dissecting cell diversity and connectivity in skeletal muscle for myogenesis
Source: Cell Death Dis. 2019 Jun 3;10(6):427. doi: 10.1038/s41419-019-1647-5 (PMC6546706; doi:10.1038/s41419-019-1647-5)

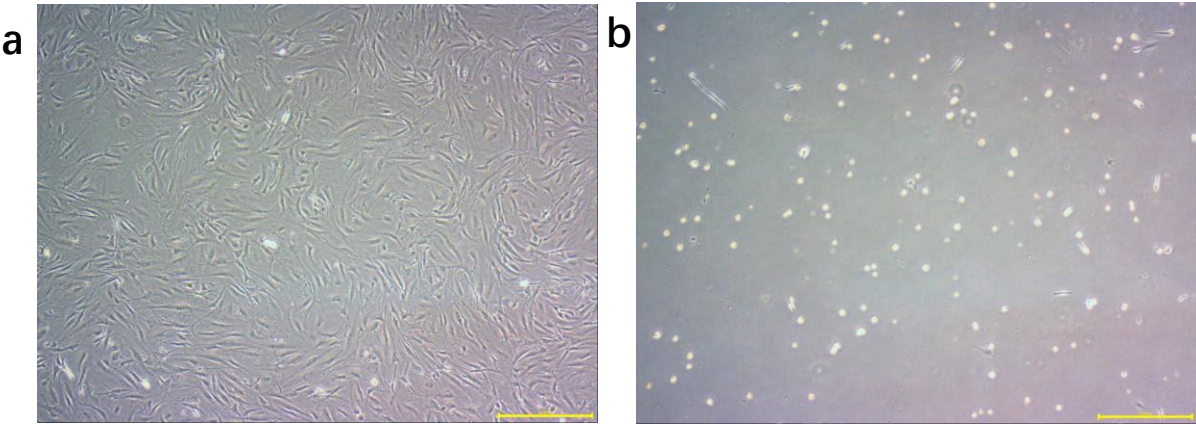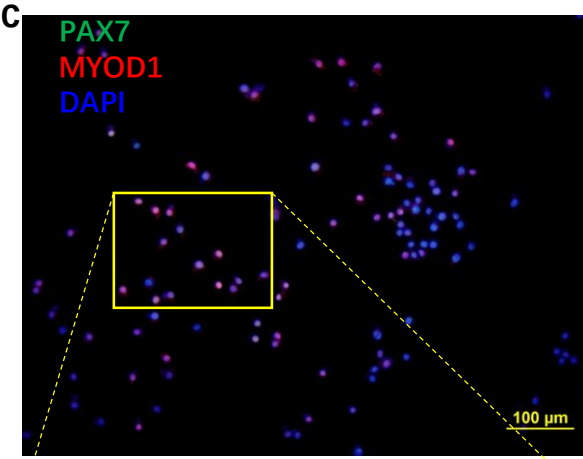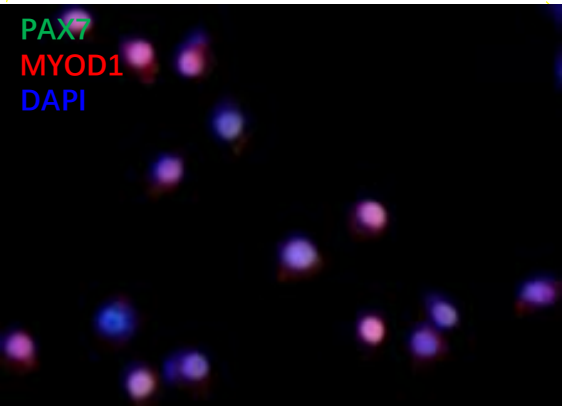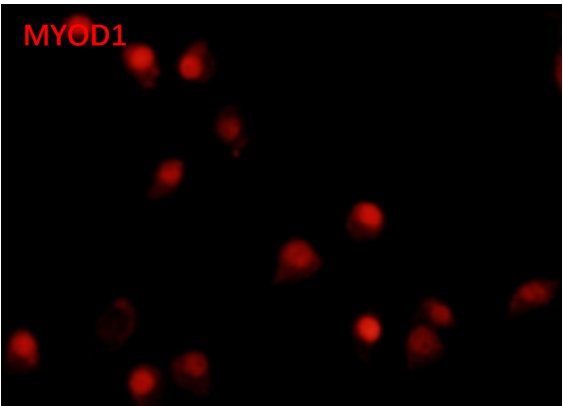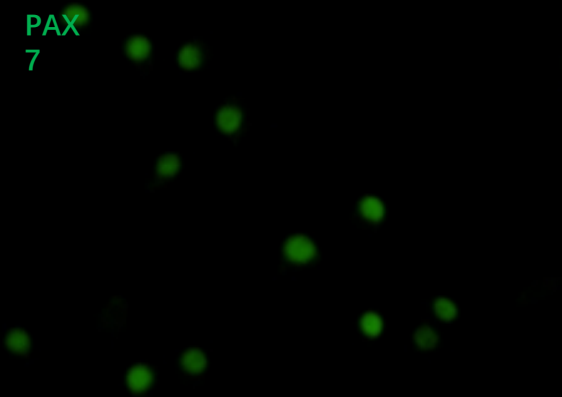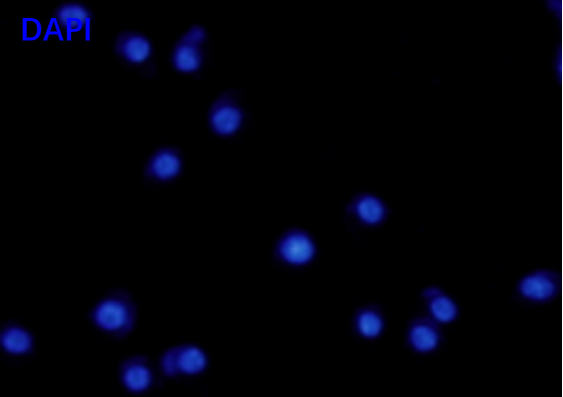

Supplement: Supplementary file 2 — Isolated RACs and MPCs [file 41419_2019_1647_MOESM2_ESM.pdf]

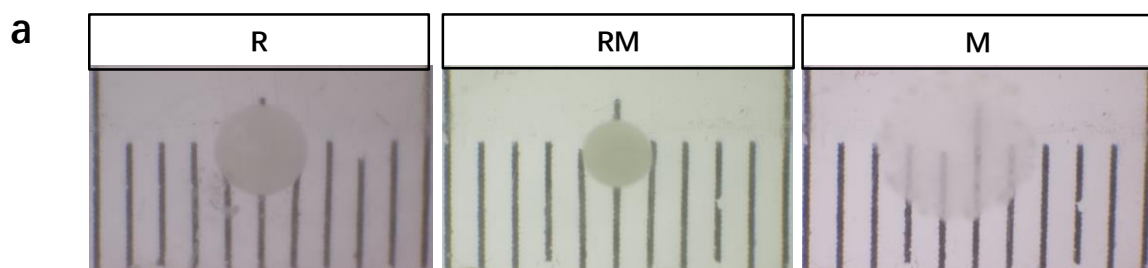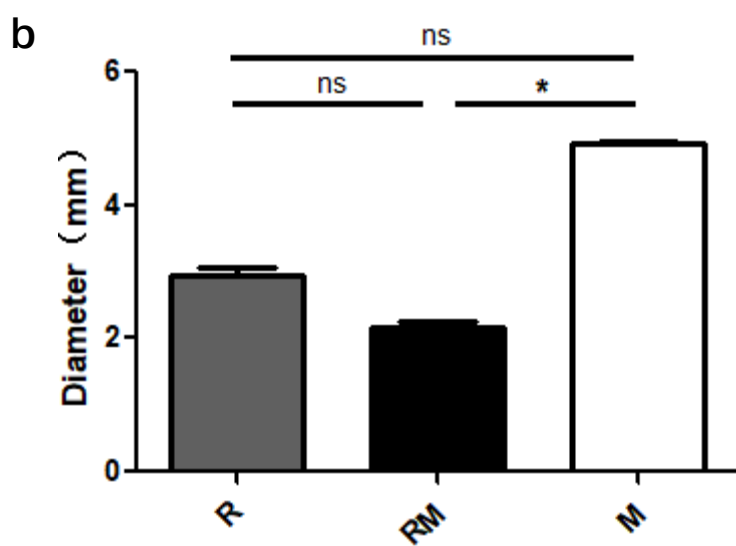

Supplement: Supplementary file 3 — Diameters of organoid after 24 h [file 41419_2019_1647_MOESM3_ESM.pdf]

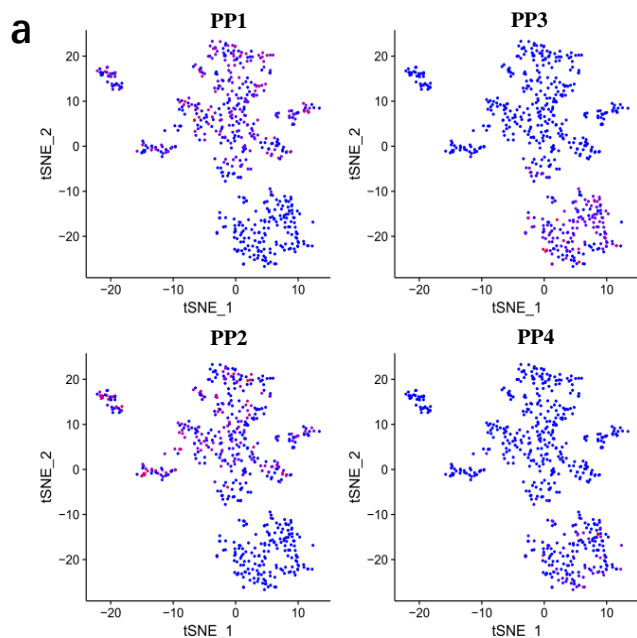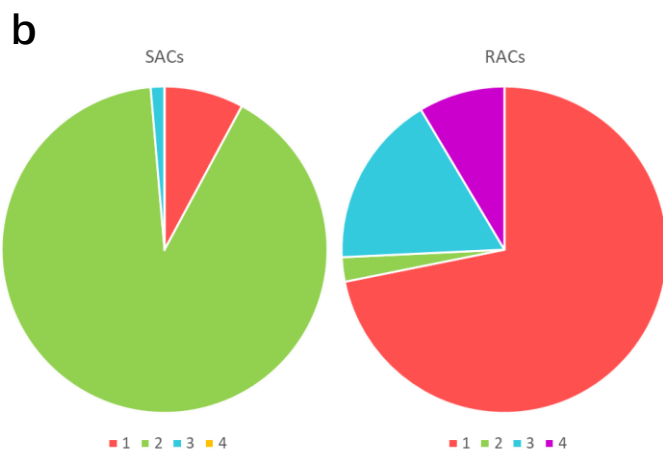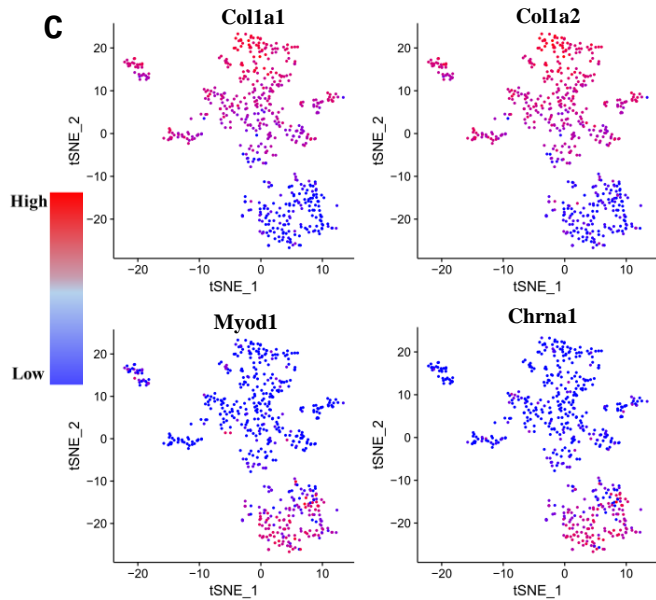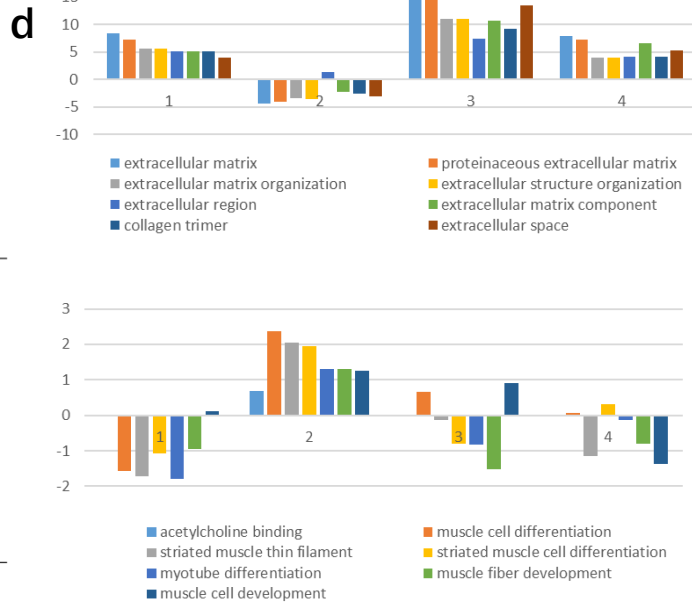

Supplement: Supplementary file 4 — Profiling characteristics of RACs and SACs with single cell RNA-seq [file 41419_2019_1647_MOESM4_ESM.pdf]

**a**

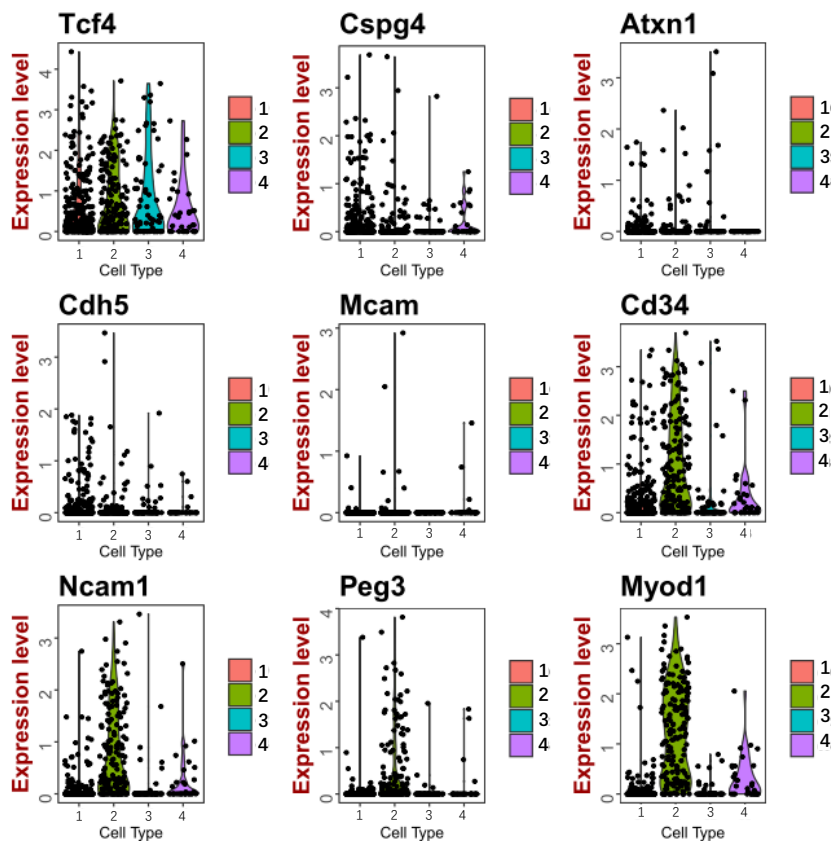

**b**

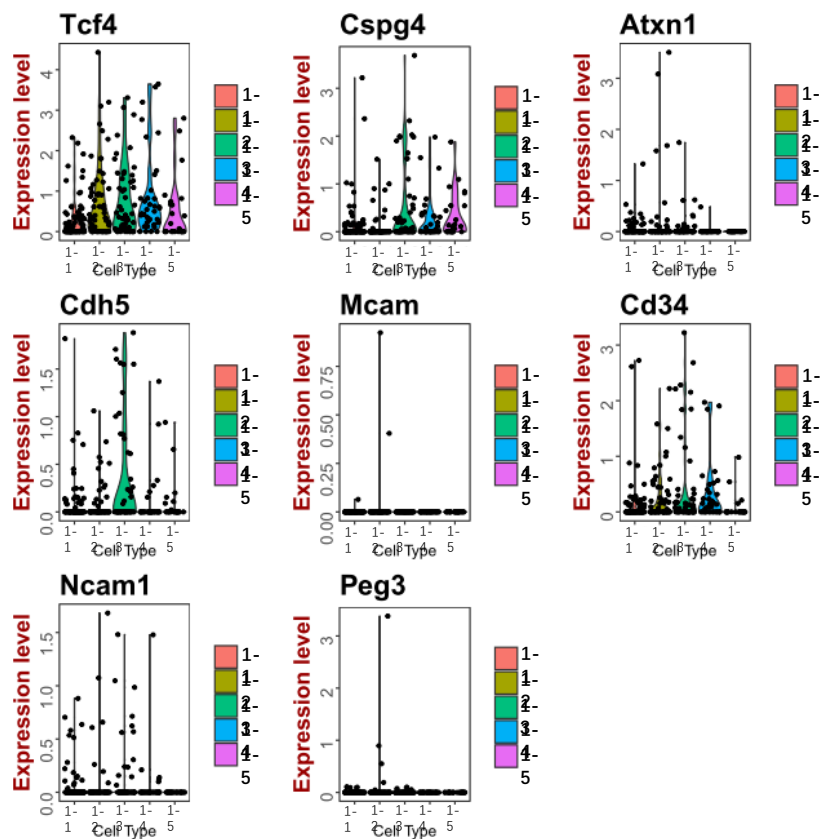

Supplement: Supplementary file 5 — The expression of presentative marker of which have been reported before in skeletal muscle derived cell types [file 41419_2019_1647_MOESM5_ESM.pdf]

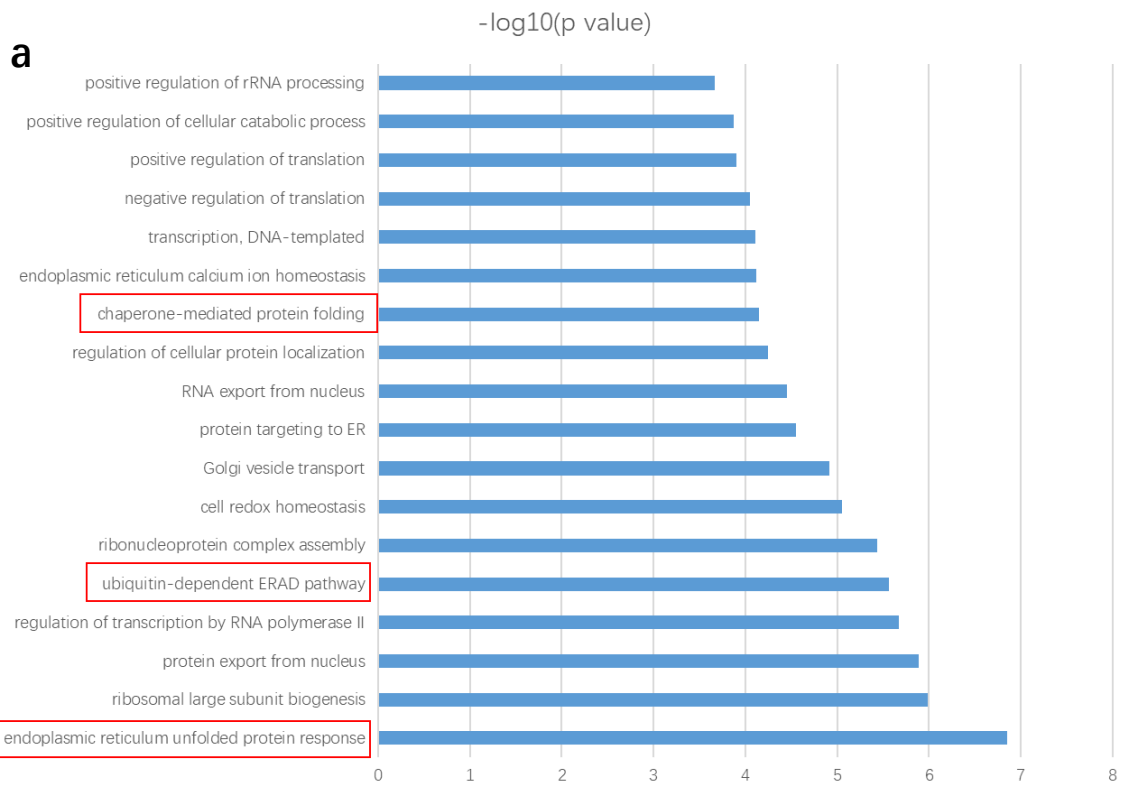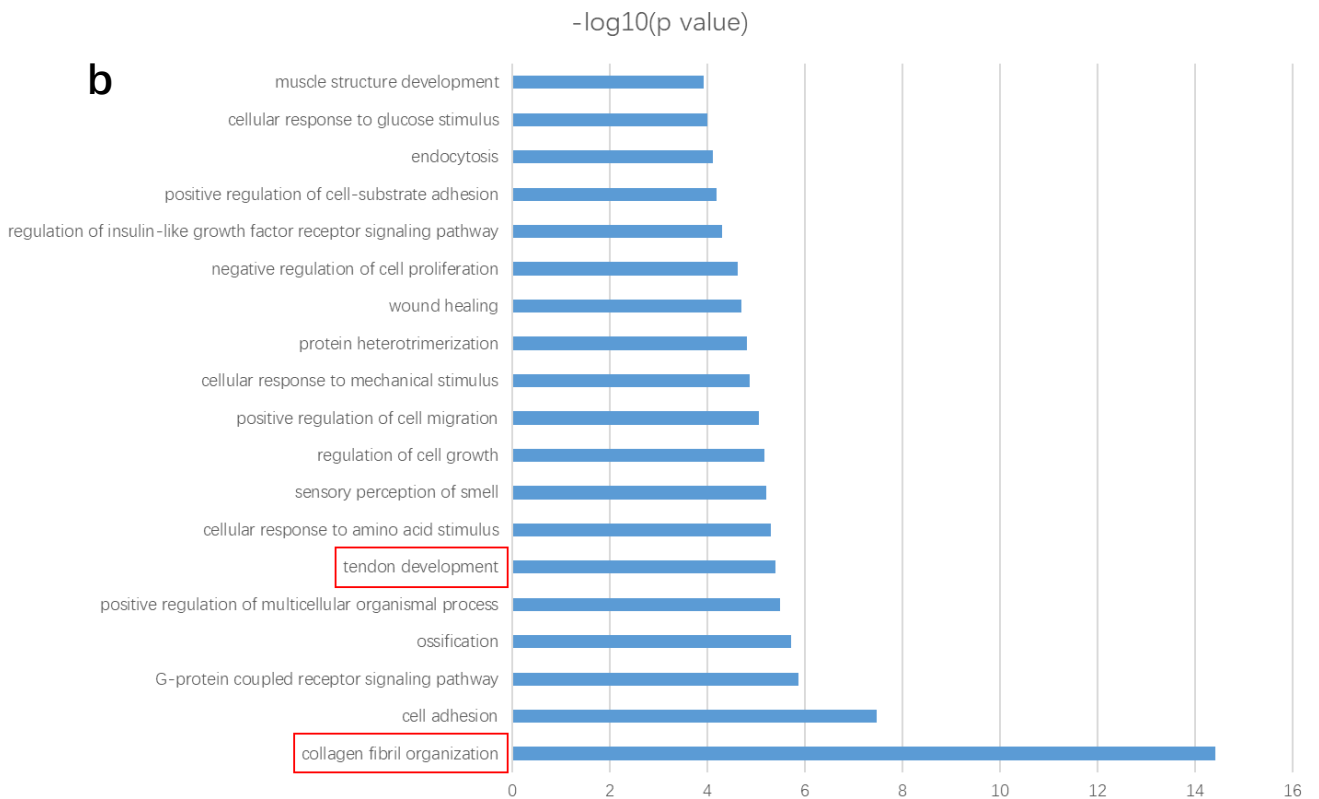

c

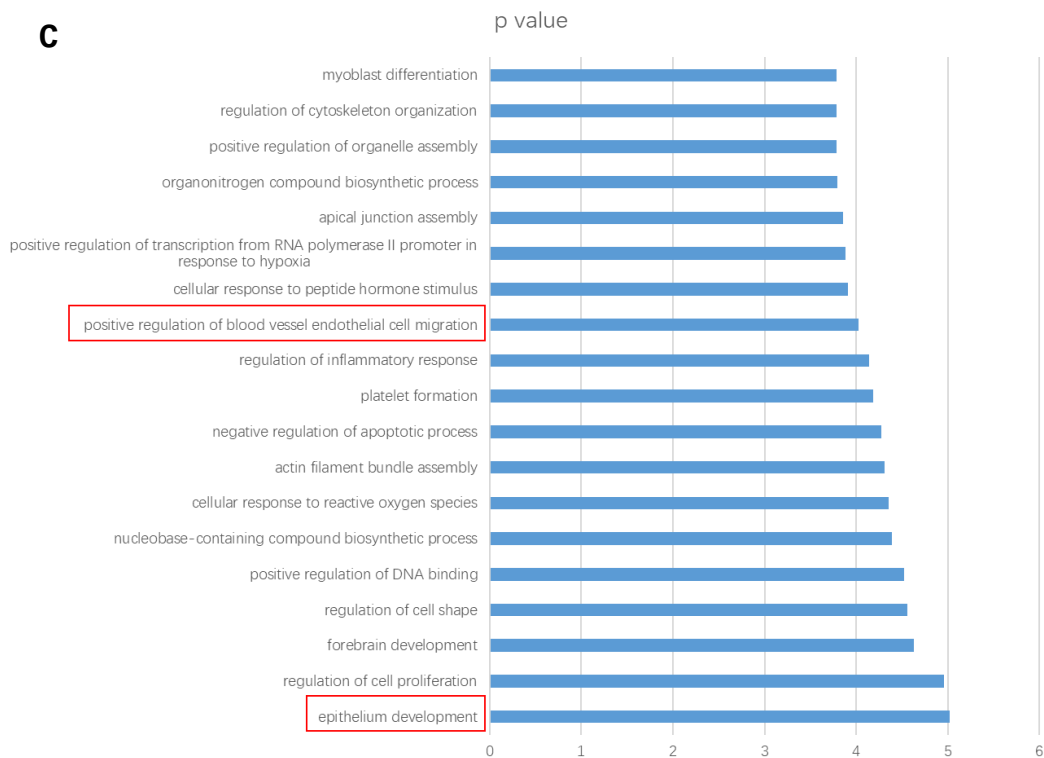

d

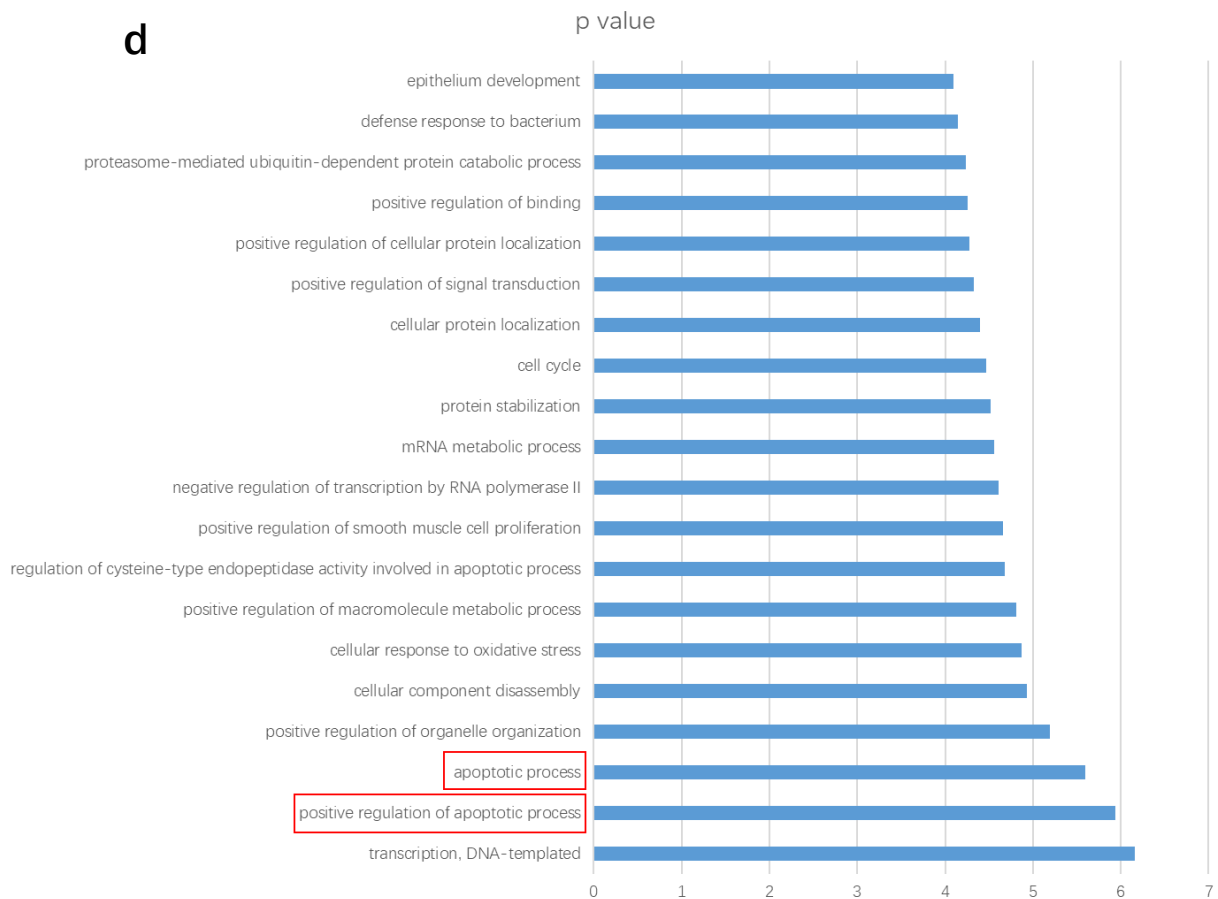

Supplement: Supplementary file 6 — The GO characteristics of C1-1,C1-2,C1-3,C1-4 [file 41419_2019_1647_MOESM6_ESM.pdf]
