# Supplementary material for: Dissecting cell diversity and connectivity in skeletal muscle for myogenesis
Source: Cell Death Dis. 2019 Jun 3;10(6):427. doi: 10.1038/s41419-019-1647-5 (PMC6546706; doi:10.1038/s41419-019-1647-5)
Supplement: Supplementary file 7 — Using the C1 High-Throughput IFC to Generate Single-cell cDNA Libraries for mRNA Sequencing [file 41419_2019_1647_MOESM7_ESM.pdf]

FLUIDIGM

PN 100-9886 C1

Using the C1 High-Throughput IFC to Generate Single-Cell cDNA Libraries for mRNA Sequencing

PROTOCOL

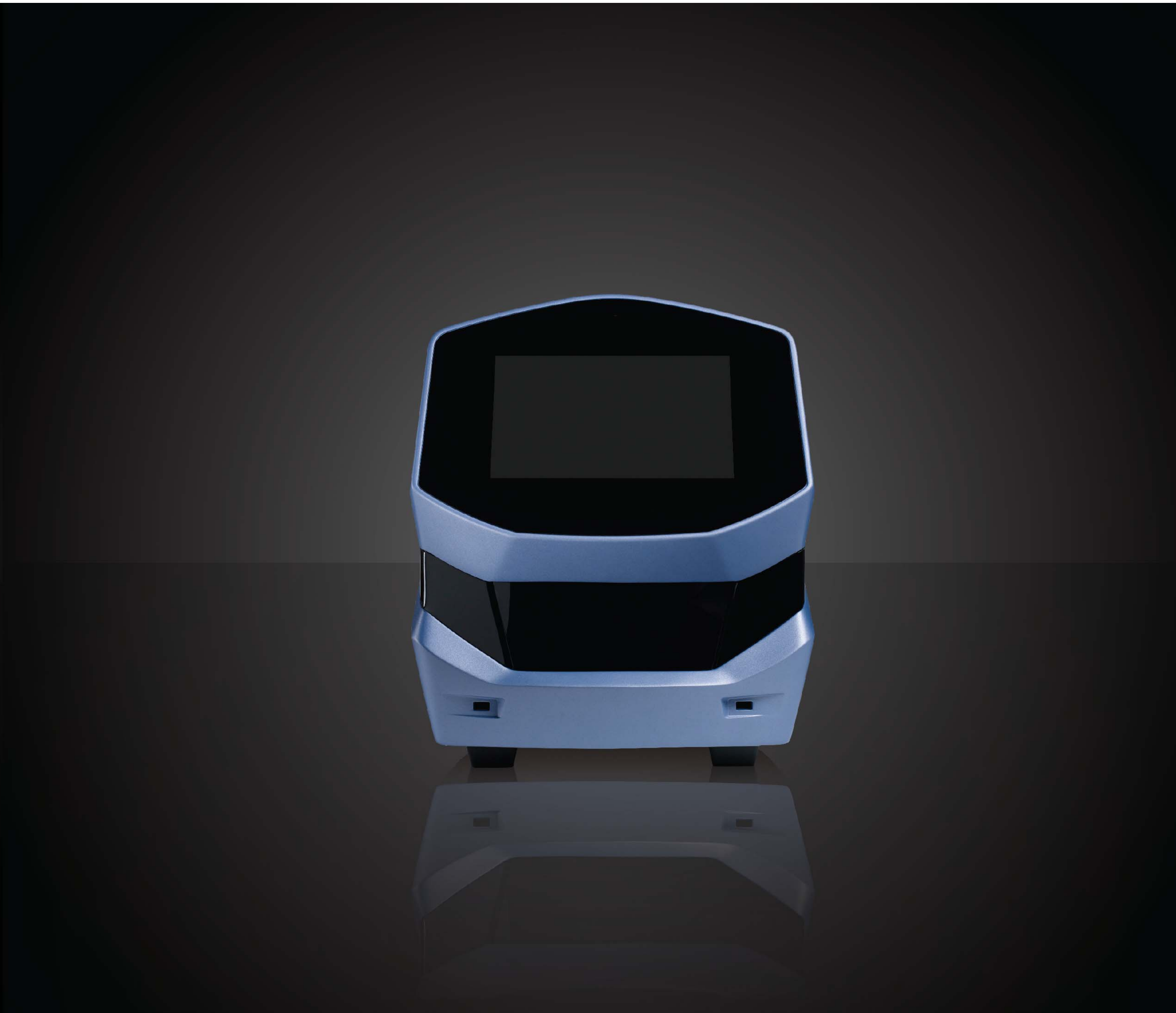

For Research Use Only. Not for use in diagnostic procedures.

Information in this publication is subject to change without notice. It is Fluidigm policy to improve products as new techniques and components become available. Therefore, Fluidigm reserves the right to change specifications at any time. Every effort has been made to avoid errors in the text, diagrams, illustrations, figures, and screen captures. However, Fluidigm assumes no responsibility for any errors or omissions. In no event shall Fluidigm be liable for any damages in connection with or arising from the use of this publication.

Patent and Limited License Information

Fluidigm products are covered by issued and pending patents in the United States and other countries. Patent and limited license information is available at fluidigm.com/legalnotices.

NOTICE TO PURCHASER:

SMART™ Amplification Products are covered by U.S. Patent Nos. 5,962,271 and 5,962,272, and corresponding foreign patents. For-Profit and Not-For-Profit purchasers of SMART Amplification Products are entitled to use the reagents for internal research. However, the following uses are expressly prohibited: (1) performing services for third parties; (2) identifying nucleic acid sequences to be included on nucleic acid arrays, blots, or in libraries or other cDNA collections which are then sold to third parties. Reproduction, modification, reformulation, or resale of the reagents provided in SMART Amplification Products is not permitted. For information on licensing SMART Technology for commercial purposes, please contact a licensing representative by phone at 650.919.7320 or by e-mail at licensing@clontech.com.

Trademarks

Fluidigm, the Fluidigm logo, C1, Script Hub, and Singular are trademarks or registered trademarks of Fluidigm Corporation in the U.S. and/or other countries. All other trademarks are the sole property of their respective owners.

For EU's WEEE directive information, go to fluidigm.com/compliance

© 2015 Fluidigm Corporation. All rights reserved. 09/2015

For technical support visit fluidigm.com/support

North America +1 650 266 6100 | Toll-free: +1 866 358 4354 in the US | techsupport@fluidigm.com

Europe +33 1 60 92 42 40 | techsupporteurope@fluidigm.com

China (excluding Hong Kong) +86 21 3255 8368 | techsupportchina@fluidigm.com

Japan +81 3 3662 2150 | techsupportjapan@fluidigm.com

All other Asian countries +1 650 266 6100 | techsupportasia@fluidigm.com

Central and South America +1 650 266 6100 | techsupportlatam@fluidigm.com

Contents

About This Guide 5
Safety Alert Conventions 5
Safety Data Sheets 6
Revision History 7

Introduction 9

Overview of mRNA Seq HT Chemistry 9

Overview of HT Cell Capture Process 12
Procedure Summary 12
Daily Workflow 13

Materials 15

Required Reagents 15
Required Reagents from Fluidigm 15
Required Reagents from Other Suppliers 16

Required Consumables 16

Required Equipment 17

Suggested Reagents 17

Suggested Consumables 17

Suggested Equipment 17

Best Practices 18

Generate High-Throughput Single-Cell cDNA Libraries for mRNA Sequencing 19

Retrieve the Reagents for cDNA Synthesis 19

Prepare Reagent Mixes 21
(Optional) ArrayControl RNA Spikes 22
Lysis Mix—Mix A 23
Lysis Mix A Plus Diluted Barcodes 24
Reverse Transcription (RT) Reaction Mix—Mix B 25
Cleanup Mix—Mix C 26
Preamplification Mix I—Mix D 26
Exonuclease Mix—Mix E 27
Preamplification Mix II—Mix F 27

Prime the HT IFC 28

Prepare and Load Cells 30
(Optional) Prepare LIVE/DEAD Cell Staining Solution 31
Prepare the Cell Mix While Priming the HT IFC 31
Load Cells 33
Image Cells 34

Run Lysis, Reverse Transcription, and Preamplification on the C1 System 35

(Optional) Start the Tube Control 36

Harvest, Clean up, and Preamplification off the C1 System 37

Prepare Libraries for Illumina Sequencing 41

Introduction 41

References 41

Retrieve the Reagents for Library Preparation 42

Quantify and Dilute Harvest Amplicons 42

Perform Tagmentation 43
Prepare cDNA for Tagmentation 43
Amplify the Tagmented cDNA 45

Pool and Clean Up the Library 47
Pool and Clean Up 47
Repeat Cleanup 49

Illumina Sequencing Requirements and Recommendations 50

Appendix A: (Optional) Run the Tube Controls 51
Introduction 51
Wash the Cell Mix 51
Perform the Tube Control Reactions 52
Dilute the Products and Process the Tube Controls 53

Appendix B: Data Analysis 55
Analysis Workflow 55

Stand-alone API Script for Demultiplexing 56
Additional Analysis Recommendations 56

Appendix C: HT IFC Pipetting Maps 57
Priming Map 57
Loading Map 58
Lysis, RT, and Cleanup Map 59
Harvesting Map 60
Column Attribution Map 61
Index Primer and Assignment Map 61

Appendix D: Library Prep Examples 62
Typical Agilent Bioanalyzer Traces 62

Appendix E: C1 Single-Cell mRNA Seq HT Reagent Kit PN 101-0063 63

Appendix F: HT IFC Types and Related Scripts 64

Appendix G: Related Documentation 65
cDNA Synthesis 65
DNA Sequencing 65

Appendix H: Safety 66
General Safety 66
Instrument Safety 66
Chemical Safety 67
Disposal of Products 67

About This Guide

CAUTION ABBREVIATED SAFETY ALERTS. Hazard symbols and hazard types specified in procedures may be abbreviated in this document. For complete safety information, see the safety appendix on page 66.

For detailed instructions on instrument and software operation, see the C1 System User Guide (PN 100-4977).

Safety Alert Conventions

This guide uses specific conventions for presenting information that may require your attention. Refer to the following safety alert conventions.

Safety Alerts for Chemicals

Fluidigm follows the United Nations Globally Harmonized System of Classification and Labelling of Chemicals (GHS) for communicating chemical hazard information. GHS provides a common means of classifying chemical hazards and a standardized approach to chemical label elements and safety data sheets (SDSs). Key elements include:

- Pictograms that consist of a symbol on a white background within a red diamond-shaped frame. Refer to the individual SDS for the applicable pictograms and warnings pertaining to the chemicals being used.

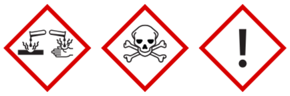

- Signal words that alert the user to a potential hazard and indicate the severity level. The signal words used for chemical hazards under GHS:

  DANGER Indicates more severe hazards.

  WARNING Indicates less severe hazards.

Safety Alerts for Instruments

For hazards associated with instruments, this guide uses the following indicators:

- Pictograms that consist of a symbol on a white background within a black triangle-shaped frame.

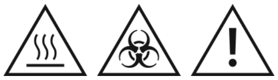

- Signal words that alert the user to a potential hazard and indicate the severity level. The signal words used for instrument hazards:

  DANGER Indicates an imminent hazard that will result in severe injury or death if not avoided.

  WARNING Indicates a potentially hazardous situation that could result in serious injury or death.

  CAUTION Indicates a potentially hazardous situation that could result in minor or moderate personal injury.

  IMPORTANT Indicates information necessary for proper use of products or successful outcome of experiments.

Safety Data Sheets

Read and understand the SDSs before handling chemicals. To obtain SDSs for chemicals ordered from Fluidigm Corporation, either alone or as part of this system, go to fluidigm.com/sds and search for the SDS using either the product name or the part number.

Some chemicals referred to in this user guide may not have been provided with your system. Obtain the SDSs for chemicals provided by other manufacturers from those manufacturers.

Revision History

Revision	Date	Description of Change
C1	23 September 2015	Various changes, including:<br>• Added the following:<br>◦ Supported sequencers (see page 9)<br>◦ Procedure summary (see page 12)<br>◦ Information on cell loading (see page 30)<br>◦ Library size distribution trace (see page 62)<br>• Updated the following:<br>◦ Replaced Life Technologies with Thermo Fisher Scientific throughout<br>◦ Clarified all steps in Figure 2 on page 11<br>◦ Clarified required reagents and consumables (see page 16)<br>◦ Removed optional tube controls from Best Practices (see page 18)<br>◦ Clarified steps to create Lysis Mix A Plus Diluted Barcodes Plate (see page 24)<br>◦ Cell imaging recommendations and guidelines (see page 34)<br>◦ Number of harvest amplicons to quantify (see page 43)
B1	25 August 2015	Changed Blocking Reagent to 10X Blocking Reagent and reduced volume to 50 µL, and changed Preloading Reagent part number to 101-1125 (see page 63).
A4	20 August 2015	Various changes, including:<br>• Added the following:<br>◦ Notice to Purchaser (see page 2)<br>◦ PBS or similar wash buffer to suggested reagents (see page 17)<br>◦ Supported sequencers (see page 41)<br>◦ Demutiplexer script download (see page 56)<br>• Updated the following:<br>◦ Process overview per day (see page 12)<br>◦ Materials lists and reagent retrieval sections, including change to Fluidigm modules (see page 15) and HT IFC part numbers (see page 16)<br>◦ Quantify and dilute harvest amplicons (see page 42)<br>◦ Cell washing steps (see page 51)<br>◦ Reagent Kit components (see page 63)<br>◦ Script names (see page 64)

Revision	Date	Description of Change
A3	7 August 2015	Various changes, including:<br>• Added the following: Introduction, Illumina Sequencing Requirements and Recommendations, Wash the Cell Mix, Appendix B: Data Analysis, Appendix E: C1 Single-Cell mRNA Seq HT Reagent Kit PN 101-0063, Appendix F: HT IFC Types and Related Scripts, and Appendix G: Related Documentation<br>• Updated the following:<br>◦ Materials lists and reagent retrieval sections, including change to sequencing kit part number (see page 16) and optional RNA spikes source (see page 17)<br>◦ Best Practices<br>◦ Required and optional procedures and figures throughout, including: clarification of reagent mix names (see page 21), renaming of lysis (see page 35) and harvest sections (see page 37)<br>• Moved the following from body of protocol to separate sections:<br>◦ Preparation of reagent mixes to Prepare Reagent Mixes and cells to Prepare and Load Cells<br>◦ Imaging of loaded cells to Image Cells<br>◦ Start of optional tube control reactions to (Optional) Start the Tube Control
A2	9 June 2015	Removed Dilution Reagent from SMARTer® Kit on page 15, added dilution of Blocking Reagent to step 8 on page 29.
A1	4 June 2015	New document on use of the new C1 HT IFC for mRNA sequencing with medium cells (10–17 µm).

Introduction

Overview of mRNA Seq HT Chemistry

This protocol details the use of the C1™ system and C1 high-throughput integrated fluidics circuits (HT IFCs) to capture up to 800 cells, apply a cell-specific barcode to all polyA+ RNA, convert polyA+ RNA into cDNA, and perform universal amplification of the cDNA for 3′ end-counting mRNA sequencing (mRNA Seq)* on Illumina® MiSeq or HiSeq systems.

The protocol explains all steps performed, including capturing cells, staining cells for viability, imaging cells, lysing cells, barcoding, performing cDNA amplification by PCR, harvesting the amplified cDNA, multiplexing the cDNA, and generating 20 sequencing-ready cDNA libraries using a modified Illumina Nextera™ XT DNA sample preparation protocol.

Figure 1 on page 10 shows how the 800 cells in the C1 mRNA Seq HT IFC are multiplexed. Cell barcodes are applied across each row of the HT IFC, and cDNA is harvested (pooled) through each of the 20 columns. Each column harvest contains barcoded cDNA from 40 cells. During library preparation, the Nextera index provides a second cell identifier, as shown in Figure 2 on page 11. Therefore, each cell is uniquely identified by a cell barcode applied on the HT IFC and a Nextera index during library preparation external to the HT IFC.

* 3′ end-counting is a technique used to determine the number of transcripts present per gene within an individual cell.

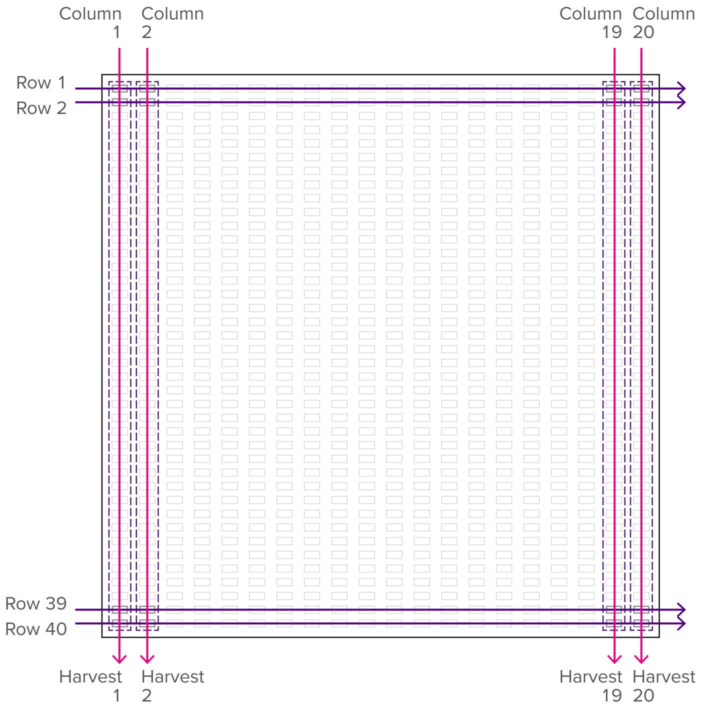

Figure 1. Overview of barcode arrangement for HT IFC harvest products (40 rows x 20 columns = 800 row-column barcode combinations)

The chemistry described in this protocol uses a modified oligo (dT) to prime first-strand cDNA synthesis by selecting for polyA+ RNA in a sample,* while simultaneously integrating a cell barcode. When the reverse transcriptase reaches the 5′ end of the mRNA, the enzyme's terminal transferase activity adds a few non-templated deoxycytidines to the 3′ end of the cDNA. The reverse transcription (RT) primer contains a few guanosines at its 3′ end that base-pair with the non-templated deoxycytidines on the cDNA to create an extended template. The reverse transcriptase extends to the end of the RT primer, producing single-stranded cDNA that contains a preamplification adapter, the 3′ end of the cDNA, and the reverse complement of the preamplification adapter. Only polyadenylated RNA containing the preamplification adapter sequence at both ends will be amplified. Lastly, sequencing adapters and Nextera indices are applied during library preparation. In this reaction, only the 3′ fragment of the transcript is enriched, therefore enabling 3′ end-counting.

* Chenchik, A., Zhu, Y.Y., Diatchenko, L. et al. "Generation and use of high-quality cDNA from small amounts of total RNA by SMART PCR," in *Gene Cloning and Analysis of RT-PCR,* BioTechniques Book (1998).

Figure 2 shows an overview of the mRNA Seq HT chemistry used in this protocol. In this 3′ end-counting approach, the cell barcode is applied across the row of the HT IFC during the reverse transcription step, while the Nextera index is used to define the column containing the cell during the 3′ end enrichment (library preparation) step:

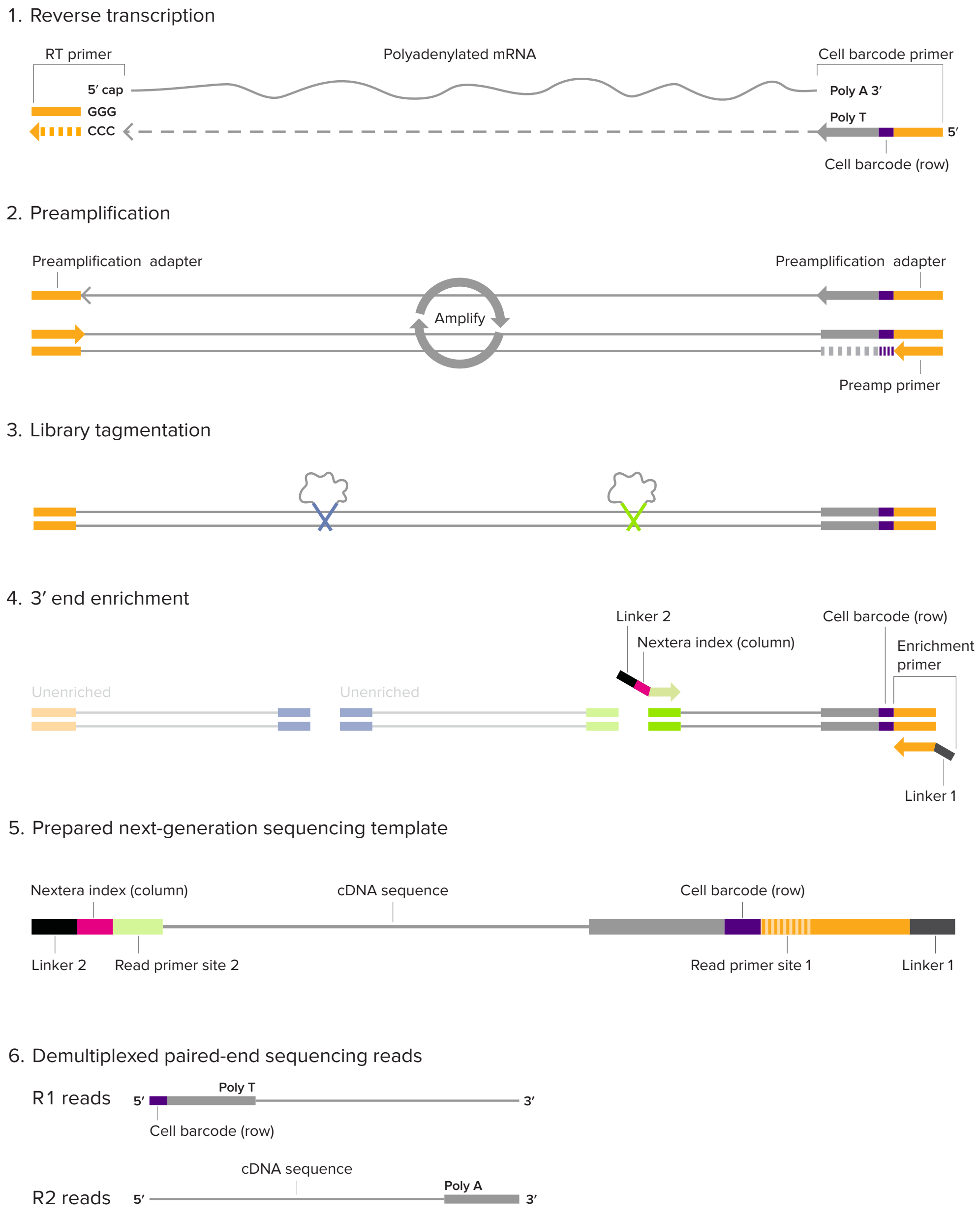

Figure 2. Overview of mRNA Seq HT chemistry

Overview of HT Cell Capture Process

Procedure Summary

During the HT cell capture process, you perform cDNA synthesis on the HT IFC and in optional tube controls, and library preparation off the HT IFC:

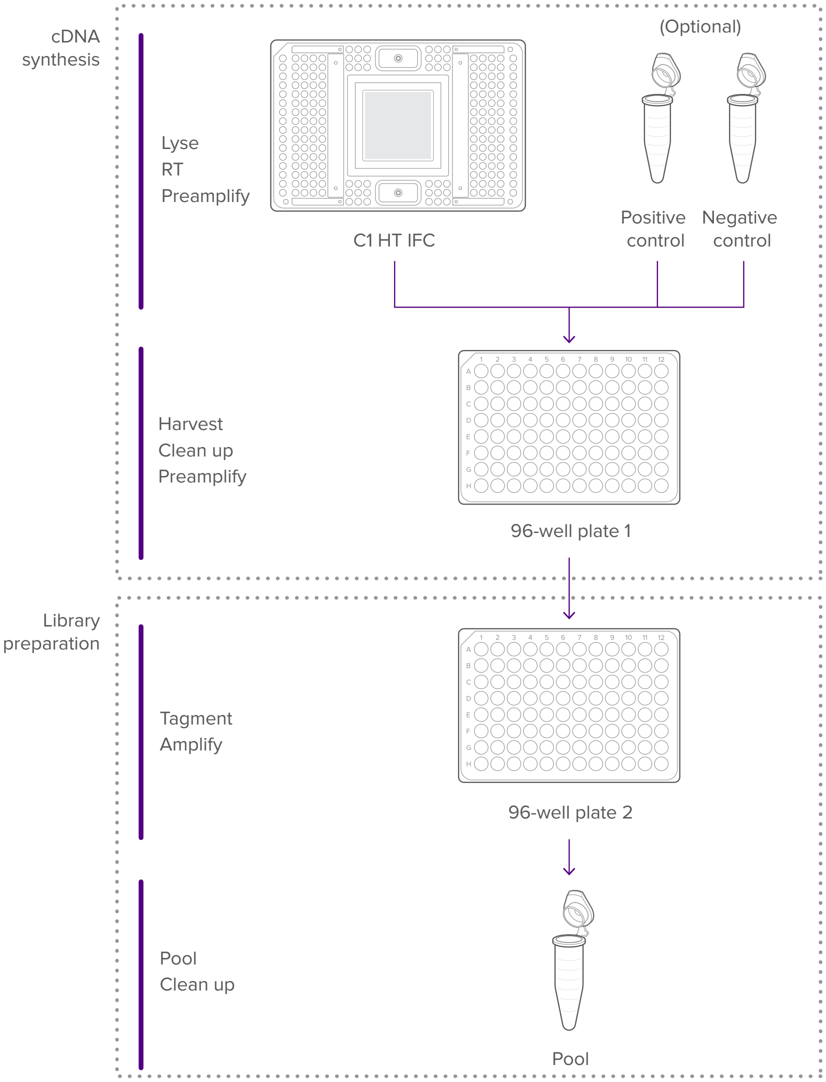

Daily Workflow

- **Day 1:** Perform lysis, RT, and preamplification on the HT IFC.
- **Day 1 or Day 2:** Perform harvest, cleanup, and preamplification off the HT IFC.
- **Day 2:** Library preparation.
- **Day 3:** mRNA sequencing.
- **Post-sequencing:** Data analysis.

NOTE

- If you are running the optional tube controls (see Appendix A on page 51), add up to 4 hours to the time estimate.
- The asterisks (*) in the workflow denote where you would incorporate the optional tube controls.

	Reagent Handling	Automated Steps	Time
**Day 1: Perform lysis, RT, and preamplification on the HT IFC**			
**1**	Prepare reagent mixes A–D.		60 min*
**2**	Pipet priming solutions into the HT IFC.		10 min
**3**		Prime the HT IFC on C1.	35 min<br>You have up to 60 minutes after the script finishes to load the HT IFC with the C1 system.
**4**	Prepare cells.		User-defined time, varies with cell type.
**5**	(Optional) Prepare viability stain.		5 min
**6**	Pipet cells into the HT IFC.		5 min*
**7**		Load cells on C1.	Medium-cell HT IFC:<br>• 40 min if staining<br>• 15 min if not staining
**8**	Image cells with a microscope.		User-defined time, varies with microscope.
**9**	Pipet lysis, reverse transcription, and preamplification chemistry into the HT IFC.		10 min
**10**		Run the mRNA sequencing script on C1. This includes lysis, reverse transcription, PCR, and harvest.	4 hours 5 min for medium-cell HT IFC*<br>You can run the script overnight with a user-defined pause between PCR and harvest functions.<br>You have up to 60 minutes after the script finishes to transfer the cDNA from the HT IFC to a plate.

	Reagent Handling	Automated Steps	Time
**Day 1 or Day 2: Perform harvest, cleanup, and preamplification off the HT IFC**			
**11**	Prepare reagent mixes E–F.		5 min
**12**	Transfer harvest amplicons from the HT IFC.		10 min*
**13**		Run the exonuclease reaction on thermal cycler.	25 min
**14**		Run preamplification on thermal cycler.	2.5 hours
**Day 2: Library preparation**			
**1**	Quantify and dilute harvest amplicons.	Run the Agilent® Bioanalyzer analysis.	60 min*
**2**	Prepare and tagment the cDNA.		30 min
**3**		Amplify the tagmented cDNA on thermal cycler.	45 min
**4**	Perform library pooling, cleanup, and quantification		60 min
**5**		Run the Agilent Bioanalyzer® analysis.	50 min
**Day 3: mRNA sequencing**			
**1**	Prepare and load samples for sequencing.	Perform sequencing on Illumina MiSeq® or HiSeq systems.	User-defined time, varies with system.
**Post-sequencing: Data analysis**			
**1**	Demultiplex and trim Illumina reads.	Perform primary analysis.	User-defined time, varies with:<br>• Number of samples<br>• Single-cell mRNA sequencing depth<br>• Server specifications and setup
**2**		Perform secondary analysis.	User-defined time [see the Singular™ Analysis Toolset User Guide (version 4.x or later)].

* Incorporate optional tube controls in this step (see Appendix A on page 51).

Materials

Required Reagents

IMPORTANT

- Store reagents as soon as they are received, according to manufacturer's storage recommendations.
- Only use the reagents provided in the required kit.
- Do not swap reagents between kits.

Required Reagents from Fluidigm

NOTE

- The C1 Single-Cell mRNA Seq HT Reagent Kit is shipped in five boxes: Module 1, Module 2, Module 3, Module 4, and Module 5.
- When ordering the modules from Fluidigm, use the kit part number: 101-0063.
- The modules in this kit provide the necessary reagents to run mRNA Seq HT chemistry on five C1 HT IFCs.
- For a diagram of the C1 Single-Cell mRNA Seq HT Reagent Kit contents (Module 1 and Module 2 reagents and volumes), see Appendix E on page 63.

Reagent	Part Number	Source	Storage	
C1 mRNA Seq HT Reagent Kit	101-0063	Fluidigm	• Module 1:	4 °C
			• Module 2:	–20 °C
			• Module 3:	–20 °C
			• Module 4:	–80 °C
			• Module 5:	–20 °C and –80 °C

Required Reagents from Other Suppliers

Reagent	Part Number	Source
SeqAmp™ DNA Polymerase	638509 (200 reactions)	Clontech
Nextera XT DNA Sample Preparation Kit (96 Samples)	FC-131-1096 (**Box 1** and **Box 2**)	Illumina
Nextera XT Index Kit v2 (96 Indices, 384 Samples)	FC-131-2001 (**Set A**) and FC-131-2002 (**Set B**)	
Agencourt® AMPure XP, 5 mL	A63880	Beckman Coulter
Exonuclease I	M0293S	New England BioLabs
Ethanol, 200 proof	–	Major laboratory supplier (MLS)
TE buffer (10 mM Tris, 1 mM EDTA; pH 8.0)	–	MLS

Required Consumables

Product	Part Number	Source
C1 Single-Cell mRNA Seq HT IFC (10–17 µm, barcode 1910x)	101-0221 (1 IFC) or 101-0222 (5 IFCs)	Fluidigm
High Sensitivity DNA Kit	5067-4626	Agilent Technologies
MAXYMum Recovery™ Microtubes (1.5 mL)	MCT-150-L-C	Axygen Scientific
MicroAmp® Clear Adhesive Film	4306311	Thermo Fisher Scientific
PCR Sealing Film	82018-844	VWR International
96-well PCR plates*	–	MLS
0.2 mL PCR 8-tube strips	–	MLS
Filtered pipette tips	–	MLS
Low-lint cloth	–	MLS

* Recommended: TempPlate® semi-skirted 96-well PCR plates (USA Scientific PN 1402-9700).

Required Equipment

Product	Part Number	Source
C1 system	100-7000	Fluidigm
2100 Bioanalyzer	G2940CA	Agilent Technologies
96-well PCR plate thermal cycler	–	MLS
Three centrifuges: one picofuge, one for microtubes, one for 96-well PCR plates	–	MLS
Vortexer	–	MLS
Magnetic stand for microtubes*	–	MLS

* Recommended: DynaMag™-2 Magnet (Thermo Fisher Scientific PN 12321D).

Suggested Reagents

Reagent	Part Number	Source
ArrayControl™ RNA Spikes	AM1780	Thermo Fisher Scientific
The RNA Storage Solution	AM7000	
LIVE/DEAD® Viability/Cytotoxicity Kit, for mammalian cells	L-3224	
1X Phosphate-buffered saline (PBS) or similar wash buffer	–	MLS

Suggested Consumables

Product	Part Number	Source
C-Chip™ Disposable Hemocytometer (Neubauer Improved)	DHC-N01	INCYTO

Suggested Equipment

Product	Part Number	Source
Two hoods (DNA and DNA-free)*	–	MLS
Imaging equipment compatible with C1 mRNA Seq HT IFCs†	–	MLS

* To prevent DNA contamination of lab and samples.
† See the Minimum Specifications for Single-Cell Imaging Specification Sheet (PN 100-5004).

Best Practices

- Use good laboratory practices to minimize contamination of samples. Use a new pipette tip for every new sample. Change gloves after handling barcodes. Whenever possible, separate pre- and post-PCR activities. Dedicate laboratory materials to designated areas.
- Thaw reagents on ice unless directed to thaw them at room temperature. Ensure that they are thawed completely and mixed before use in a reaction. Mix and centrifuge reagents as directed. Do not vortex reagents unless directed. Avoid creating bubbles.
- To ensure reliable results, we recommend that you do the following:
  - Establish a working cell dissociation protocol for each new cell type you will run on the HT IFC before proceeding with this protocol.
  - Practice microscopy imaging before loading the HT IFC for the first time. If you practice with an unused IFC, cover the IFC with PCR sealing film to prevent contamination during imaging.
  - Use the pipetting maps provided with this protocol (see Appendix C on page 57 for all maps in a printable format).
- When pipetting into the HT IFC:
  - Ensure that the notch (A1 position) is at the top-left corner of the HT IFC and the barcode faces to the left.
  - Always stop at the first stop on the pipette to avoid creating bubbles in the inlets. If a bubble is introduced, ensure that it floats to the top of the well.
  - Make sure to evenly distribute reagents over the bottom surfaces of the accumulators and reservoirs.

Generate High-Throughput Single-Cell cDNA Libraries for mRNA Sequencing

Retrieve the Reagents for cDNA Synthesis

IMPORTANT To ensure reliable results, follow the Best Practices on page 18.

NOTE

- For a diagram of the C1 Single-Cell mRNA Seq HT Reagent Kit contents (Module 1 and Module 2 reagents and volumes), see Appendix E on page 63.
- The modules in this kit provide the necessary reagents to run mRNA Seq HT chemistry on five C1 HT IFCs. Retrieve only the reagents required from each module based on the number of HT IFCs you will run.

Retrieve the following reagents only when needed in your daily workflow (see page 12):

		Required Reagent		Preparation	Source
1	HT IFC priming (see page 28)	C1 Harvest Reagent	●	Remove from –20 °C and thaw to room temperature in a DNA-free hood	C1 HT Kit, Module 2
		Preloading Reagent	●		
		Valve Fluid	●	Remove from 4 °C and equilibrate to room temperature in a DNA-free hood	C1 HT Kit, Module 1
		10X Blocking Reagent	●		
		Stability Solution	●		
		Cell Rinsing Reagent	○		
2	(Optional) RNA Spikes Mix (see page 22)	ArrayControl RNA Spikes		Remove from –80 °C, thaw on ice, and keep on ice	ArrayControl RNA Spikes
		The RNA Storage Solution		Keep at room temperature	The RNA Storage Solution
3	(Optional) RNA Spikes Mix dilution (see page 23)	RNA Spikes Mix		Remove aliquot from –80 °C and thaw on ice if stored, and keep on ice	From workflow step 2
		RNase Inhibitor		Remove from –20 °C, thaw on ice, and keep on ice	C1 HT Kit, Module 5
		Preloading Reagent	●	Remove from –20 °C and thaw to room temperature in a DNA-free hood	C1 HT Kit, Module 2

		Required Reagent	Preparation	Source
**4**	Lysis Mix—Mix A (see page 23)	(Optional) RNA Spikes Mix dilution	Remove aliquot from –80 °C, thaw on ice, and keep on ice	From workflow step 3
		10X Lysis Buffer - v3	Remove from –20 °C, thaw on ice, and keep on ice	C1 HT Kit, Module 5
		RNase Inhibitor		
		Nuclease-Free Water	Remove from –20 °C and thaw to room temperature	
**5**	Lysis Mix A plus diluted barcodes (see page 24)	Cell Barcode Plate	Remove from –20 °C, thaw on ice, and keep on ice	C1 HT Kit, Module 3
		Lysis Mix A	Keep on ice	From workflow step 4
		TE buffer	Keep at room temperature	User-supplied
**6**	Reverse Transcription Mix—Mix B (see page 25)	Reverse Transcription Primer	Remove from –80 °C, thaw on ice, and keep on ice	C1 HT Kit, Module 4
		Loading Reagent	Remove from –20 °C and thaw to room temperature	C1 HT Kit, Module 2
		Nuclease-Free Water	Remove from –20 °C and thaw to room temperature	C1 HT Kit, Module 5
		5X First-Strand Buffer	Remove from –20 °C, thaw on ice, and keep on ice	
		Dithiothreitol (DTT)		
		SMARTer dNTP Mix (20 mM each)		
		RNase Inhibitor		
		SmartScribe™ Reverse Transcriptase	Remove from –20 °C just before use	
**7**	Cleanup Mix—Mix C (see page 26)	10X Exonuclease I Reaction Buffer	Remove from –20 °C and thaw to room temperature	Exonuclease I
		C1 Harvest Reagent	Remove from –20 °C and thaw to room temperature	C1 HT Kit, Module 2
**8**	(Optional) LIVE/DEAD cell staining (see page 31)	Ethidium homodimer-1	Remove from –20 °C and thaw to room temperature (keep in the dark as much as possible)	LIVE/DEAD Kit
		Calcein AM		
		Cell Rinsing Reagent	Remove from 4 °C and equilibrate to room temperature	C1 HT Kit, Module 1
**9**	HT IFC loading (see page 30)	Cell mix	Prepare, count, and resuspend to appropriate concentration	User-supplied
		Cell Rinsing Reagent	Remove from 4 °C and equilibrate to room temperature	C1 HT Kit, Module 1
		Suspension Reagent	Remove from 4 °C and vortex well	

		Required Reagent		Preparation	Source
**10**	HT IFC lysis (see page 35)	Preloading Reagent	● (purple)	Remove from –20 °C and thaw to room temperature	C1 HT Kit, Module 2
**11**	Exonuclease Mix—Mix E (see page 27)	Exonuclease I (*E. coli*)		Remove from –20 °C and keep on ice	Exonuclease I
		Cleanup Mix—Mix C		Remove from 4 °C if stored overnight and keep on ice until use	From workflow step 7
**12**	Preamplification Mix—Mix D, Mix F (see pages 26 and 27)	SeqAmp PCR Buffer (2X)		Remove from –20 °C, thaw on ice, and keep on ice	SeqAmp DNA Polymerase
		SeqAmp DNA Polymerase		Remove from –20 °C just before use	
		Loading Reagent	● (yellow)	Remove from –20 °C and thaw to room temperature	C1 HT Kit, Module 2
		Preamp Primer	● (blue)	Remove from –20 °C, thaw on ice, and keep on ice	
**13**	Cell mix wash for optional tube controls (see page 51)	1X PBS (or similar wash buffer)		Keep at room temperature	User-supplied

Prepare Reagent Mixes

IMPORTANT To ensure reliable results, follow the Best Practices on page 18.

Prepare the following reagent mixes only when needed in your daily workflow (see page 12):

Reagent Mix	When to Prepare
• (Optional) ArrayControl RNA Spikes on page 22<br>• Lysis Mix—Mix A on page 23<br>• Lysis Mix A Plus Diluted Barcodes on page 24<br>• Reverse Transcription (RT) Reaction Mix—Mix B on page 25	Day 1
Cleanup Mix—Mix C on page 26	Day 1 (can also store overnight at 4 °C if you program to harvest on Day 2)
Preamplification Mix I—Mix D on page 26	Day 1
Exonuclease Mix—Mix E on page 27	Day 1 (or optionally Day 2)
Preamplification Mix II—Mix F on page 27	Day 1 (or optionally Day 2)

(Optional) ArrayControl RNA Spikes

RNA spikes serve as a positive control for thermal cycling of the C1 system that is independent of cell capture. Although this control is not required, it is highly recommended.

NOTE

- Due to the low volume pipetted, we highly recommend making the ArrayControl RNA Spikes mix in bulk and aliquoting for future use.
- ArrayControl RNA Spikes contain eight RNA transcripts. We will use only three.

Prepare the RNA Spikes Mix

1. After the ArrayControl RNA Spikes have thawed, remove spikes 1, 4, and 7 from the box.
2. Combine the following reagents in three separate microtubes:

Tube	A	B	C
The RNA Storage Solution	13.5 µL	12.0 µL	148.5 µL
RNA Spikes	**No. 7:** 1.5 µL	**No.4:** 1.5 µL	**No. 1:** 1.5 µL

3. Vortex tube A for 3 seconds and centrifuge to collect contents. Pipet 1.5 µL from tube A into tube B. Discard tube A.
4. Vortex tube B for 3 seconds and centrifuge to collect contents. Pipet 1.5 µL from tube B into tube C. Discard tube B.
5. Vortex tube C for 3 seconds and centrifuge to collect contents. Tube C is the concentrated RNA standard that may be aliquoted and frozen for future use.
6. Aliquot 1.25 µL into separate microtubes and store at −80 °C until use. One tube is necessary for each C1 run.

Dilute the RNA Spikes Mix for the Lysis Mix (Mix A)

NOTE Diluted RNA does not store well. Do not dilute RNA more than an hour before you load the HT IFC. Only store concentrated aliquots long term.

1 Thaw an RNA Spikes Mix aliquot (from step 6 above).

2 Dilute by combining:

Table 1. RNA Spikes Mix dilution

Reagent	Volume (µL)
Preloading Reagent (Fluidigm)	28.7
RNAse Inhibitor (Fluidigm)	1.0
RNA Spikes Mix aliquot (from step 6 above)	1.0
**Total**	**30.7**

3 Vortex for 3 seconds and centrifuge briefly to collect contents.

Lysis Mix—Mix A

Prepare the Lysis Mix (Mix A) before loading cells, or immediately before use in lysis, RT, and preamplification on the HT IFC. You can prepare Lysis Mix with or without the optional RNA spikes.

1 Combine the following reagents in a microtube labeled “A.”

Components	Volume (without RNA spikes; µL)	Volume (with RNA spikes; µL)
10X Lysis Buffer - v3 (Fluidigm: **do not vortex**)	38.0	38.0
RNase Inhibitor (Fluidigm)	2.0	2.0
Nuclease-Free Water (Fluidigm)	172.0	161.4
(Optional) RNA Spikes Mix dilution (from Table 1)	—	10.6
**Total**	**212.0**	**212.0**

2 Gently pipet up and down a few times to mix. **Avoid creating bubbles**. Centrifuge briefly to collect contents. Keep on ice until use.

Lysis Mix A Plus Diluted Barcodes

You must first prepare a diluted barcodes plate, then prepare a plate containing Lysis Mix A plus the diluted barcodes:

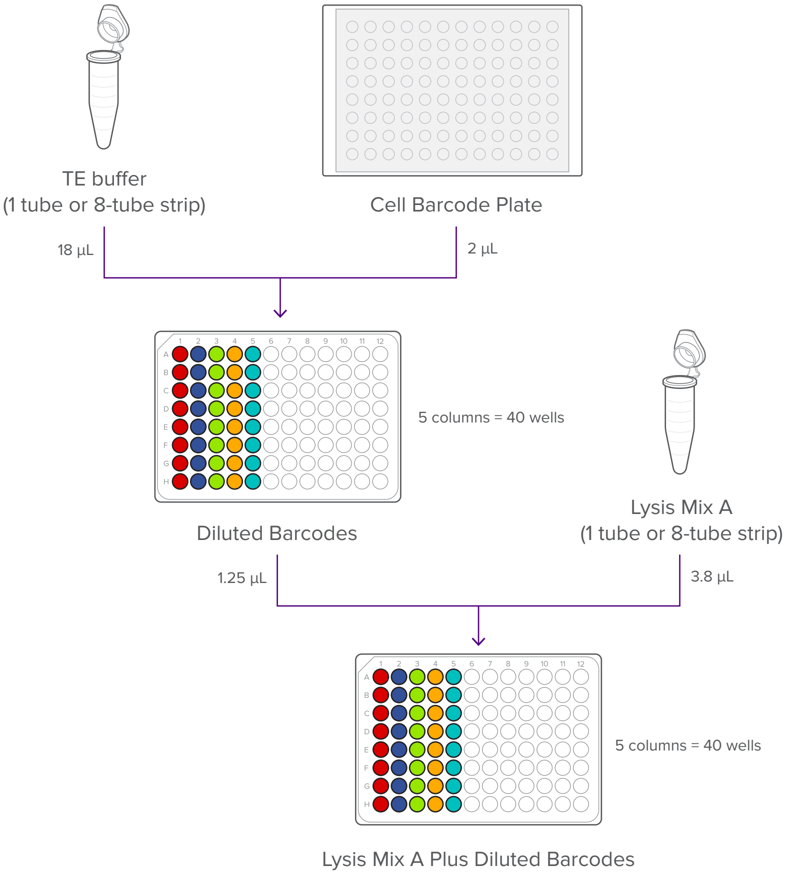

1 To prepare the diluted barcode plate:

a Centrifuge the Cell Barcode Plate at ≈ 3,000 x *g* for 2 minutes (**do not vortex**).

b Aliquot 100 µL of TE buffer into each tube of an 8-tube strip, then use a multichannel pipette to pipet 18 µL of TE buffer into the first 5 columns of a 96-well PCR plate labeled “Diluted Barcodes” (40 wells total).

c Using a multichannel pipette with fresh P200 pipette tips for each well, pierce the foil on the Cell Barcode Plate, then pipet 2 µL of the barcodes column by column into the same 40 wells of the Diluted Barcodes plate. Pipet up and down a few times to mix. **Pipet carefully to avoid contamination.** Keep the Diluted Barcodes plate on ice until use in step 2b.

2 To prepare a plate containing Lysis Mix A plus diluted barcodes:

a Pipet 3.8 µL of the Lysis Mix A into the first 5 columns of a new 96-well PCR plate labeled “Lysis Mix A Plus Diluted Barcodes” (40 total wells). **Pipet carefully to avoid bubbles**.

NOTE
- You can aliquot 23 µL of Lysis Mix A into each tube of an 8-tube strip for ease of pipetting.
- (Optional) If performing a tube control (see Appendix A on page 51), pipet 3.8 µL of the Lysis Mix A into 2 microtubes.

b Avoiding bubbles, carefully pipet 1.25 µL of the diluted barcodes from the Diluted Barcodes plate column by column into the 40 wells of the Lysis Mix A Plus Diluted Barcodes plate from step 2a, using fresh pipette tips for each column.

NOTE (Optional) If performing a tube control, randomly select 2 barcodes and pipet 1.25 µL of the selected barcodes into the 2 microtubes from step 2a.

c Cover the Lysis Mix A Plus Diluted Barcodes plate with PCR sealing film. Centrifuge the plate at ≈ 3,000 x *g* for 2 minutes (**do not vortex**). Keep on ice until use.

Reverse Transcription (RT) Reaction Mix—Mix B

Prepare the RT Reaction Mix (Mix B) before loading cells, or immediately before use in lysis, RT, and preamplification on the HT IFC.

1 Combine the following reagents in a microtube labeled "B."

Components		Volume (µL)
5X First-Strand Buffer (RNase-free) (Fluidigm)		13.6
Dithiothreitol (DTT) (Fluidigm)		1.7
SMARTer dNTP Mix (20 mM each) (Fluidigm)		3.4
Reverse Transcription Primer (Fluidigm)	● (red)	3.4
RNase Inhibitor (Fluidigm)		1.7
Nuclease-Free Water (Fluidigm)		4.3
Loading Reagent (Fluidigm)	● (yellow)	1.2
Mix, then add (**do not vortex**): SMARTScribe Reverse Transcriptase (Fluidigm)		6.8
**Total**		**36.1**

2 Gently pipet up and down a few times to mix. **Avoid creating bubbles**. Centrifuge briefly to collect contents. Keep on ice until use.

Cleanup Mix—Mix C

Prepare the Cleanup Mix (Mix C) before loading cells, or immediately before use in lysis, RT, and preamplification on the HT IFC.

1 Combine the following reagents in a microtube labeled "C."

Table 2. Cleanup Mix

Components		Volume (µL)
10X Exonuclease I Reaction Buffer (New England BioLabs)		39
C1 Harvest Reagent (Fluidigm)	●	244
**Total**		**283**

2 Gently vortex and centrifuge briefly to collect contents. Keep on ice until use.

NOTE If you program to harvest the next day (see page 36), you can store Cleanup Mix C and remaining C1 Harvest Reagent overnight at 4 °C until you are ready to harvest.

Preamplification Mix I—Mix D

Prepare the Preamplification Mix I (Mix D) before loading cells, or immediately before use in lysis, RT, and preamplification on the HT IFC.

1 Combine the following reagents in a microtube labeled "D."

Components		Volume (µL)
SeqAmp PCR Buffer (2X) (Clontech)		150.0
Loading Reagent (Fluidigm)	●	5.1
Preamp Primer (Fluidigm)	●	1.5
Mix (**do not vortex**), then add: SeqAmp DNA Polymerase (Clontech)		6.0
**Total**		**162.6**

2 Gently pipet up and down a few times to mix. **Avoid creating bubbles**. Centrifuge briefly to collect contents. Keep on ice until use.

Exonuclease Mix—Mix E

Prepare Exonuclease Mix (Mix E) only immediately before use in harvest, cleanup, and preamplification off the HT IFC.

Combine the following reagents in a microtube labeled “E.”

Components	Volume (µL)
Cleanup Mix C (see Table 2)	39
Exonuclease I Enzyme (New England BioLabs)	1
**Total**	**40**

IMPORTANT Concentration of the Exonuclease I Enzyme is extremely important. Be very careful to ensure that no droplets are stuck to the pipette tip when pipetting, to avoid introducing additional enzyme to Cleanup Mix C.

Preamplification Mix II—Mix F

Prepare Preamplification Mix II (Mix F) immediately before use in harvest, cleanup, and preamplification off the HT IFC.

1 Combine the following reagents in a microtube labeled “F.”

Components		Volume (µL)
SeqAmp PCR Buffer (2X) (Clontech)		133.0
Loading Reagent (Fluidigm)	● (yellow)	4.5
Preamp Primer (Fluidigm)	● (blue)	1.33
Mix (**do not vortex**), then add: SeqAmp DNA Polymerase (Clontech)		5.3
**Total**		**144.13**

2 Gently pipet up and down a few times to mix. **Avoid creating bubbles**. Centrifuge briefly to collect contents. Keep on ice until use.

Prime the HT IFC

IMPORTANT To ensure reliable results, follow the Best Practices on page 18.

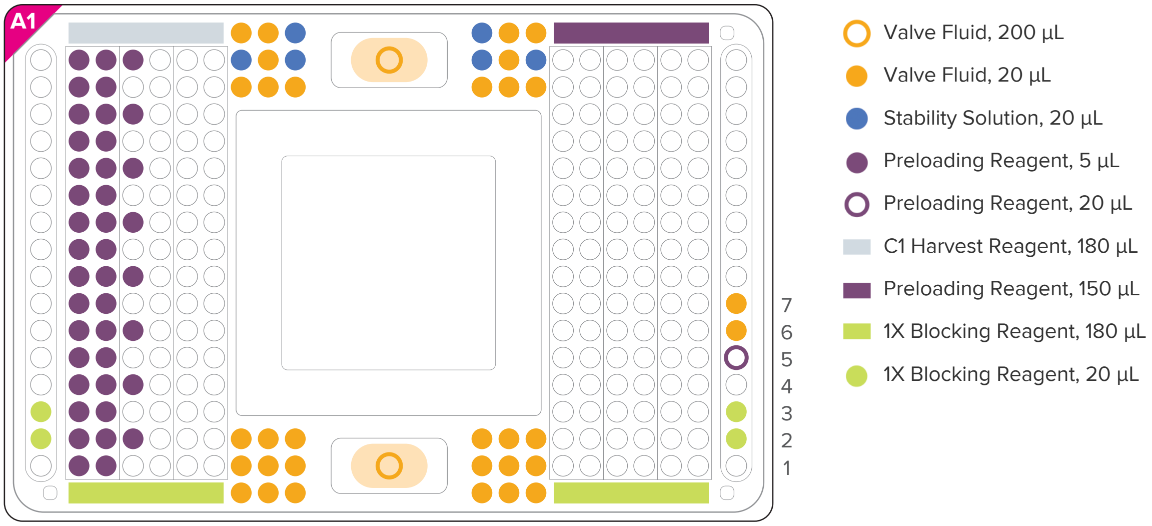

Figure 3. HT IFC priming pipetting map

1. Pipet 200 µL of Valve Fluid into each of the accumulators marked with gold outlined circles (, see Figure 3). The middle of the bottom surface should be immersed ( ).

   IMPORTANT To ensure optimal results, the Valve Fluid must be distributed evenly over the bottom surface of both accumulators ( ). Gently tap the HT IFC on the bench to distribute the fluid over the bottom surface.

2. Pipet 20 µL of Valve Fluid into each of the 30 control line inlets near the accumulators and the hydration inlets 6–7, all marked with solid gold circles ( ).

3. Using a P200 pipette, slowly pipet 20 µL of Stability Solution into each of the 6 inlets marked with blue circles ( ).

   IMPORTANT The Stability Solution is viscous. Make sure to use a P200 pipette. Pipet the solution slowly and carefully to avoid bubbles. The final volume transferred to each well may be less than 20 µL.

4. Aliquot 30 µL of Preloading Reagent into each tube of an 8-tube strip, then use a multichannel pipette to pipet 5 µL of Preloading Reagent into each of the 40 barcode inlets marked with solid purple circles ( ).

5. Pipet 20 µL of Preloading Reagent into well 5, marked with a purple outlined circle ( ).

6. Pipet 150 µL of Preloading Reagent into the PCR reservoir at the top right of the HT IFC marked with a solid purple rectangle ( ).

7 Pipet 180 µL of C1 Harvest Reagent into the harvest reservoir at the top left of the HT IFC marked with a solid gray rectangle ( ).

8 Dilute the 10X Blocking Reagent to 1X:

Components		Volume (µL)
10X Blocking Reagent (Fluidigm)	●	50
Cell Rinsing Reagent (Fluidigm)	○	450
**Total**		**500**

9 Pipet 180 µL of 1X Blocking Reagent into each of the 2 wash reservoirs at the bottom of the HT IFC marked with solid light green rectangles ( ).

10 Pipet 20 µL of 1X Blocking Reagent into each of the 4 stain and cell inlets marked with solid light green circles (●).

11 Peel off white tape on bottom of HT IFC, then place the IFC into the C1 system. Run the **mRNA Seq HT: Prime (1910x)** script. Priming takes approximately 32 minutes. When the Prime script has finished, tap **EJECT** to remove the primed IFC from the instrument.

NOTE

- During HT IFC priming, prepare the cell mix for loading (see Prepare and Load Cells on page 30).
- After priming the HT IFC, you have up to 1 hour to load the IFC with the C1 system.
- See the C1 System User Guide (PN 100-4977) for instructions for use.

Prepare and Load Cells

IMPORTANT To ensure reliable results, follow the Best Practices on page 18.

The center panel of the HT IFC is divided into two sections, left and right, with 10 columns of 40 capture sites in each section. You can load the same or different cell mixes into each section through the corresponding cell inlets:

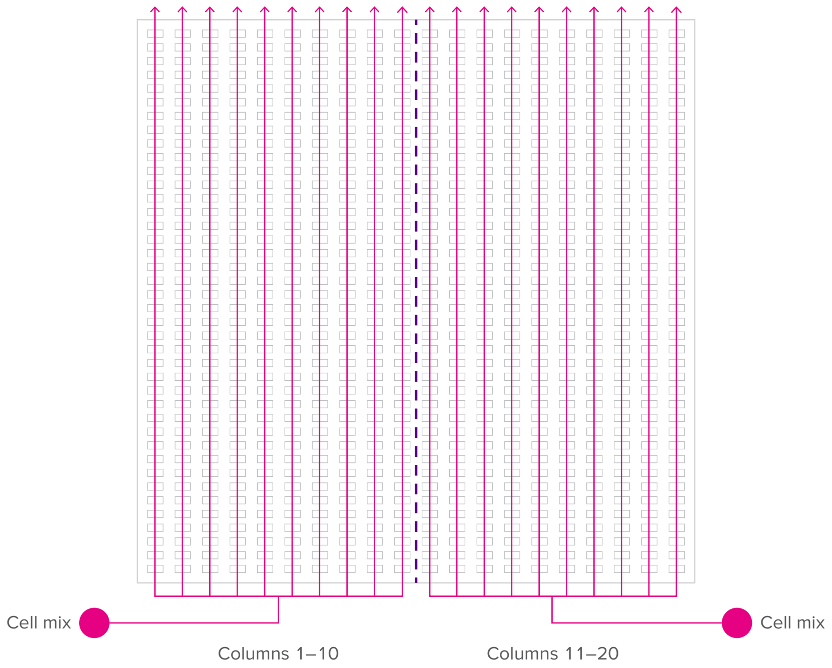

Figure 4. Cell loading pattern in the center panel of the HT IFC

Follow these protocols to prepare the cell mix for loading into the HT IFC:

- (Optional) Prepare LIVE/DEAD Cell Staining Solution on page 31
- Prepare the Cell Mix While Priming the HT IFC on page 31
- Load Cells on page 33
- (Optional) Start the Tube Control on page 36
- Image Cells on page 34

(Optional) Prepare LIVE/DEAD Cell Staining Solution

The LIVE/DEAD Viability/Cytotoxicity Kit tests the viability of a cell based on the integrity of the cell membrane. This test contains two chemical dyes. The first dye is green-fluorescent calcein AM, which stains live cells. This dye is cell-permeable and tests for active esterase activity in live cells. The second dye is red-fluorescent ethidium homodimer-1, which will stain cells only if the integrity of the cell membrane has been lost.

IMPORTANT

- Keep the dye tubes closed and in the dark as much as possible, because they can hydrolyze over time. When not in use, store in dark, airtight bag with desiccant pack at −20 °C.
- Cell staining solution may be prepared up to 2 hours before loading into the HT IFC. Keep on ice and protected from light before pipetting into the IFC.

NOTE Staining medium (10–17 µm) cells takes 40 minutes.

1 Vortex the dyes for 10 seconds and then centrifuge them before pipetting.

2 Prepare the LIVE/DEAD staining solution by combining reagents in this order:

Components	Volume (µL)
Cell Rinsing Reagent (Fluidigm)	1,250.0
Ethidium homodimer-1 (Thermo Fisher Scientific)	2.5
Calcein AM (Thermo Fisher Scientific)	0.625
**Total**	**1,253.125**

3 Vortex the LIVE/DEAD staining solution well before pipetting into the HT IFC.

Prepare the Cell Mix While Priming the HT IFC

IMPORTANT Vortex the Suspension Reagent for 5 seconds before use. If Suspension Reagent contains particulates, ensure they are properly removed by vortexing. **Do not vortex** the cells.

1 Ensure that you have begun priming the HT IFC (see Prime the HT IFC on page 28).

2 Before mixing cells with Suspension Reagent and loading them into the HT IFC, prepare a cell suspension of 200–400 cells/µL in native medium using an established protocol for your cell type(s).

NOTE

- Cells may be counted by any preferred method. If an established cell counting protocol does not exist, we suggest using the disposable hemocytometer C-Chip by INCYTO. See incyto.com/product/product02_detail.php for instructions for use.
- Make sure to record your final cell concentration.
- The ratio of cells to Suspension Reagent may need to be optimized to maximize cell capture. [See the Fluidigm Single-Cell Preparation Guide (PN 100-7697).]
- You can load 1 or 2 cell mixes into the HT IFC.

3 Prepare cell mix by combining cells with Suspension Reagent at a ratio of 3:2, or as determined for your cell type(s). For example:

Components		Volume (µL)
Suspension Reagent (Fluidigm)	●	40
Cells (200–400/µL)		60
**Total**		**100**

NOTE The volume of cell mix may be scaled depending on volume of cells available. A minimum volume of 12 µL of cell mix is necessary for each cell inlet in the HT IFC.

a Pipet 40 µL of Suspension Reagent to a microtube.

b Gently and slowly add 60 µL of cells to the Suspension Reagent.

c Gently pipet the cell mix up and down 5–10 times to mix, depending on whether the cells tend to clump. **Do not vortex**. **Avoid creating bubbles**.

Load Cells

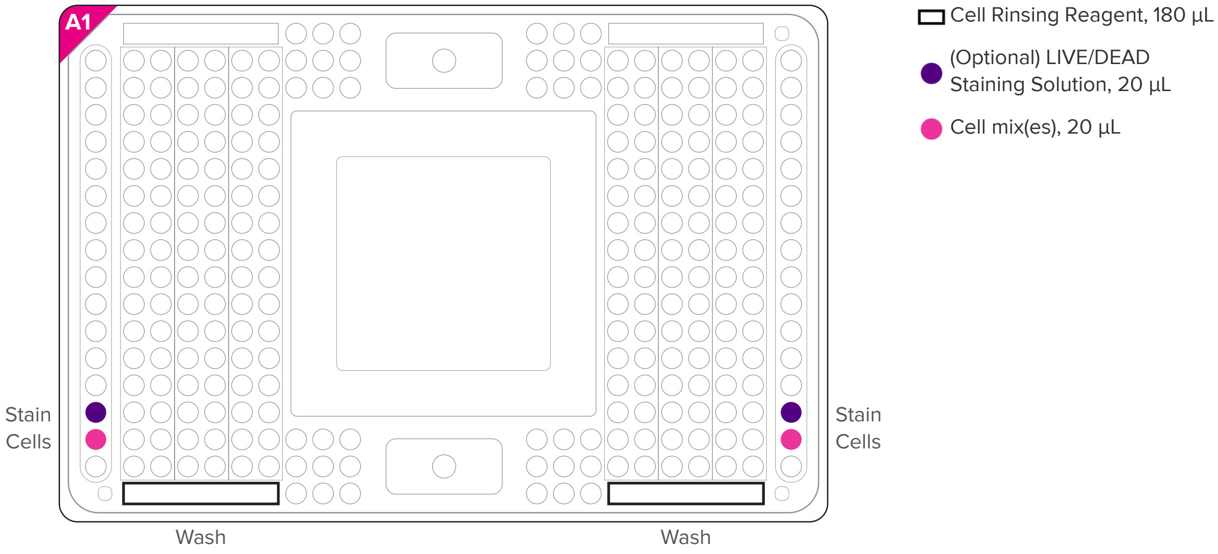

Figure 5. HT IFC loading pipetting map

1 Remove 1X Blocking Reagent from the 2 wash reservoirs, 2 cell inlets, and 2 stain inlets.

2 Add 180 µL of Cell Rinsing Reagent to each of the 2 bottom wash reservoirs marked with black outlined rectangles (▭) as shown in Figure 5.

3 Perform one of these tasks:

- **Staining cells:** Vortex the LIVE/DEAD staining solution well, then pipet 20 µL of the solution into each of 2 stain wells marked with solid purple circles (●).
- **Not staining cells:** Pipet 20 µL of Cell Rinsing Reagent into each of 2 stain wells marked with solid purple circles (●).

NOTE Staining medium (10–17 µm) cells takes 40 minutes.

4 Set a P200 pipette to 60 µL, then pipet the cell mix up and down 5–10 times to mix, depending on whether the cells tend to clump. **Do not vortex**. **Avoid creating bubbles**.

5 Pipet 20 µL of the cell mix into one or both of the cell inlets marked with solid magenta circles (●).

NOTE

- You may pipet up to 20 µL of cell mix, but only 12 µL will enter each cell inlet in the HT IFC.
- (Optional) If performing a tube control, keep the remaining cell mix on ice for later use in the tube control reactions (see Wash the Cell Mix on page 51).

6 Place the HT IFC into the C1 system. Run the **mRNA Seq HT: Cell Load (1910x)** or **mRNA Seq HT: Cell Load & Stain (1910x)** script. This script is approximately 36 minutes with staining and approximately 10 minutes without staining.

7 When the script has finished, tap **EJECT** to remove the HT IFC from the C1 system.

Image Cells

We recommend that you image all of the cell capture sites in the center panel of the HT IFC using a microscope compatible with IFCs, and then perform quality control (scoring) on the cells using an established protocol for your cell type(s). Alternatively, you can choose to image cell capture sites in a subset of columns or rows as long as they span both left and right sections of the HT IFC (see Figure 4 on page 30).

NOTE See the following for additional imaging guidelines:

- Selection of a microscope – Minimum Specifications for Single-Cell Imaging (PN 100-5004).
- Viewing cell capture sites – Using a Microscope with an Automated Stage Quick Reference (PN 100-6130).
- Quality control of cells – Fluidigm Single-Cell Preparation Guide (PN 100-7697).

Run Lysis, Reverse Transcription, and Preamplification on the C1 System

IMPORTANT To ensure reliable results, follow the Best Practices on page 18.

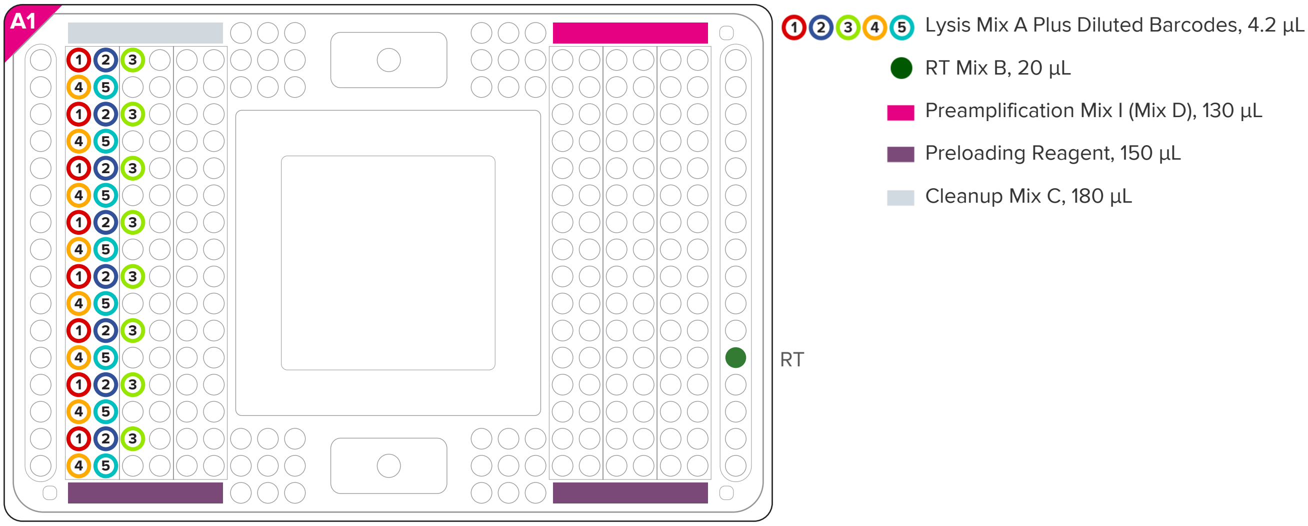

Figure 6. HT IFC lysis, RT and cleanup pipetting map

1 If necessary, prepare the following reagent mixes:
   - Lysis Mix—Mix A (see page 23)
   - Lysis Mix A Plus Diluted Barcodes (see page 24)
   - Reverse Transcription (RT) Reaction Mix—Mix B (see page 25)
   - Cleanup Mix—Mix C (see page 26)
   - Preamplification Mix I—Mix D (see page 26)

2 Remove all reagents from the 40 barcode inlets, from all 4 corner reservoirs, and from the RT inlet.

3 Very carefully transfer, column by column, 4.2 µL of the Lysis Mix A containing diluted barcodes (see page 24) into each of the corresponding wells marked 1–5 in Figure 6 (①②③④⑤). Use fresh pipette tips for each column.

4 Pipet 20 µL of RT Mix B (see page 25) into the RT well marked with a solid green circle (●).

5 Pipet 180 µL of Cleanup Mix C (see page 26) to the harvest reservoir marked with a solid gray rectangle (▬).

   NOTE If you program to harvest the next day (see step 8), you can store remaining Cleanup Mix C overnight at 4 °C until you are ready to harvest.

6 Pipet 150 µL of Preloading Reagent into each of the 2 bottom wash reservoirs marked with solid purple rectangles (▬).

7 Pipet 130 µL of Preamplification Mix I (Mix D, see page 26) into the top right reservoir marked with a solid magenta rectangle (▬).

8 Place the HT IFC into the C1 system and immediately run the **mRNA Seq HT: RT & Amp (1910x)** script. The script takes approximately 4 hours and 5 minutes to complete.

STOPPING POINT This protocol can be programmed to harvest at a convenient time. Slide the orange box (end time) to the desired time. For example, the harvest function could be programmed to next morning. Make sure to store any required reagents or optional tube controls as directed elsewhere in this protocol.

NOTE To abort the harvest, tap **ABORT**. The HT IFC will no longer be usable. Start a new experiment with a new IFC.

(Optional) Start the Tube Control

If you are running tube controls, see Appendix A on page 51 for instructions. We recommend that you start to run the tube control reaction after you start the mRNA Seq HT: RT & Amp (1910x) script.

Harvest, Clean up, and Preamplification off the C1 System

IMPORTANT To ensure reliable results, follow the Best Practices on page 18.

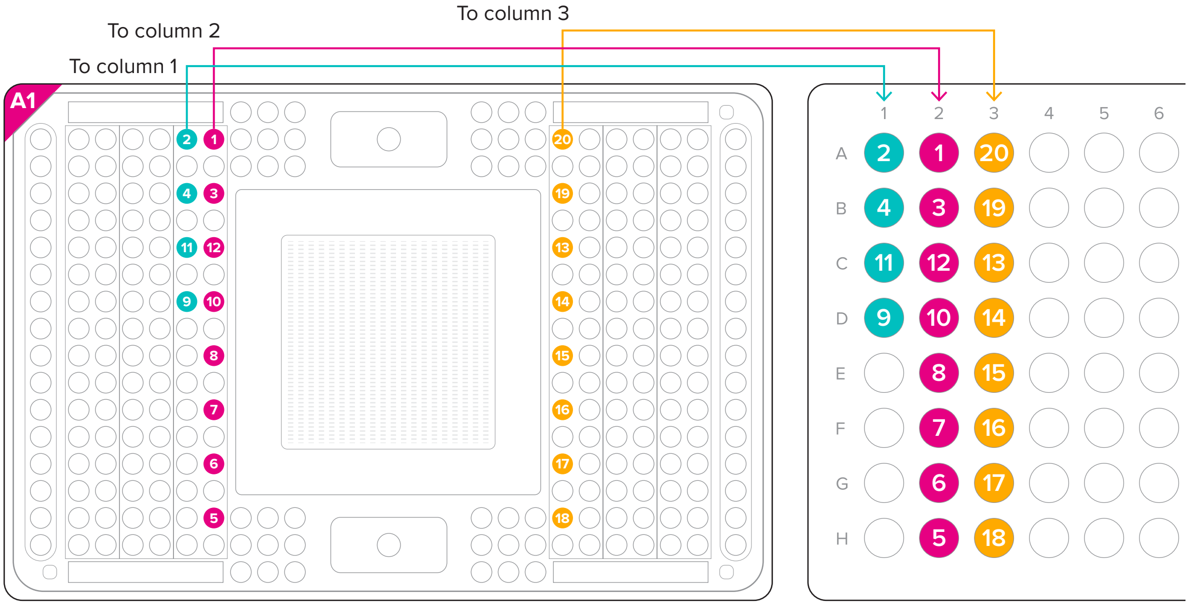

Figure 7. HT IFC harvest pipetting map

1 If necessary, prepare the following reagent mixes:
   - Exonuclease Mix—Mix E (see page 27)
   - Preamplification Mix II—Mix F (see page 27)

2 When the mRNA Seq HT: RT & Amp (1910x) script has finished, tap **EJECT** to remove the HT IFC from the instrument.

   NOTE The HT IFC may remain in the C1 system for up to 1 hour after harvest before you remove products from their inlets.

3 Transfer the HT IFC to a post-PCR lab environment.

4 Set up the following exonuclease thermal cycling protocol:

Temperature	Time	Cycles
37 °C	Preheat and pause	
37 °C	30 sec	1
80 °C	20 min	1
4 °C	Hold	1

IMPORTANT To ensure optimal results, the thermal cycler must be preheated to 37 °C before starting the protocol.

5 Carefully pull back the tape covering the harvesting inlets on the left and right side of the HT IFC using the plastic removal tool:

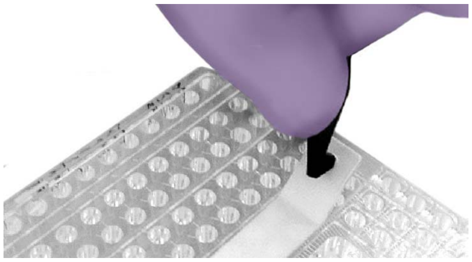

6 Using a multichannel pipette set to 5 µL, transfer the harvested amplicons from each column of inlets and into the corresponding wells of a 96-well PCR plate (see Figure 7).

NOTE .

- The volume harvested from each well may be less than 5 µL
- (Optional) If performing a tube control, transfer the diluted product from the tube control preamplification reaction (see Dilute the Products and Process the Tube Controls on page 53) into the appropriate well(s) of the harvest plate, then continue with the protocol.

7 Pipet 1 µL of the Exonuclease Mix E (see page 27) into each harvest well as in step 6.

8 Seal the harvest plate, centrifuge it at ≈ 3,000 x *g* for 1 minute to collect harvest products, then immediately place the plate in the preheated thermal cycler and run the protocol (see step 4).

IMPORTANT To ensure optimal results, the protocol must be started within 1 minute of centrifuging the plate.

9 When the thermal protocol completes, immediately place the plate on ice.

10 Add the following to the corresponding wells of a clean 96-well PCR plate (see Figure 8):

a **Slowly** pipet 5.6 µL of Preamplification Mix II (Mix F, see page 27) into each well.

IMPORTANT The Preamplification Mix II is a viscous solution. Pipet the mix slowly and carefully to avoid bubbles.

b Pipet 5 µL of the exonuclease-treated samples into each well, vortex briefly, centrifuge at ≈ 3,000 x *g* for 1 minute, then keep on ice.

NOTE (Optional) If performing a tube control (see Appendix A on page 51), pipet 5 µL of each tube control reaction in the recommended wells.

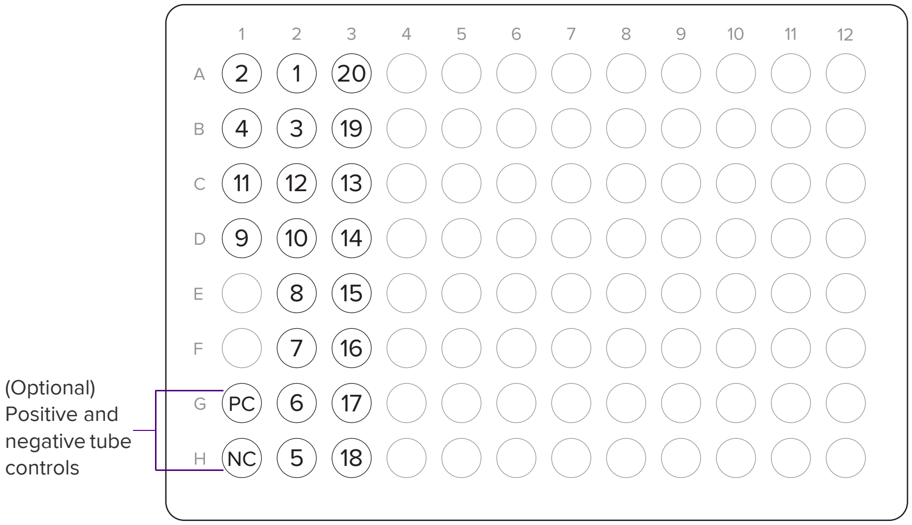

Figure 8. HT IFC column attribution to plate harvest position

11 Set up the following preamplification protocol:

Temperature	Time	Cycles
96 °C	Preheat and pause	
96 °C	1 min	1
98 °C	20 sec	1
59 °C	4 min	
68 °C	6 min	
98 °C	20 sec	9
65 °C	30 sec	
68 °C	6 min	
98 °C	30 sec	7
65 °C	30 sec	
68 °C	7 min	
72 °C	10 min	1
4 °C	Hold	

IMPORTANT To ensure optimal results, the thermal cycler must be preheated to 96 °C before starting the protocol.

12 Place the harvest plate in the preheated thermal cycler, then run the protocol.

STOPPING POINT The samples are now ready for library preparation for sequencing (see Prepare Libraries for Illumina Sequencing on page 41). You can store the harvest amplicons at 4 °C overnight on the thermal cycler, then proceed to Quantify and Dilute Harvest Amplicons on page 42.

Prepare Libraries for Illumina Sequencing

IMPORTANT To ensure reliable results, follow the Best Practices on page 18.

Introduction

Perform the modified Illumina Nextera XT DNA library preparation protocol for single-cell mRNA sequencing on MiSeq or HiSeq® systems using cDNA acquired from the HT IFC.

IMPORTANT The Illumina Nextera XT DNA Library Preparation Guide provides detailed instructions for library preparation; however, modifications have been made in order to adapt the Nextera XT chemistry to the single-cell mRNA sequencing application. We highly recommend that you carefully read the Nextera XT DNA Library Preparation Guide to familiarize yourself with the basic concepts and handling instructions before proceeding with this modified protocol.

NOTE From here, each diluted column harvest from the HT IFC is referred to as a "sample."

References

- Illumina Nextera XT DNA Library Preparation Guide
- Agilent 2100 Bioanalyzer 2100 Expert User's Guide

Retrieve the Reagents for Library Preparation

Retrieve the following reagents only when needed in your daily workflow (see page 12):

Required Reagents	Preparation	Source
DNA Dilution Reagent	Remove from −20 °C and thaw to room temperature in a DNA-free hood	C1 HT Kit, Module 2
C1 Harvest Reagent		
Enrichment Primer		
Nextera XT Index Primers:* • **Set A:** N701–N707, N710–N712, and N714–N715 • **Set B:** N716, N718–N724, and N726–N729	Remove from −20 °C and thaw to room temperature in a DNA-free hood	Nextera XT Index Kit v2
Amplicon Tagment Mix	Remove from −20 °C and keep on ice	Nextera XT Kit, Box 1
Tagment DNA Buffer		
Nextera PCR Master Mix (NPM)		
Neutralize Tagment (NT) Buffer	Remove from 4 °C and equilibrate to room temperature in a DNA-free hood	Nextera XT Kit, Box 2
Agencourt AMPure® XP	Remove from 4 °C and equilibrate to room temperature in a DNA-free hood	Beckman Coulter
High Sensitivity DNA Reagents	Remove from 4 °C and equilibrate to room temperature in a DNA-free hood	Agilent Technologies
Ethanol, 200 proof	Keep at room temperature	Major laboratory supplier (MLS)

* Only 20 of the 24 total indices are required for use (see page 46).

Quantify and Dilute Harvest Amplicons

IMPORTANT To ensure reliable results, follow the Best Practices on page 18.

cDNA concentrations yielded from the C1 system may vary with cell types and cell treatments. Library yield and size distribution also vary with input cDNA/DNA concentrations. To minimize library prep variation and to achieve high library quality, carefully determine the harvest concentration and dilution.

1 Run an Agilent High Sensitivity DNA Chip to check for yield and quality of cDNA (see Figure 10 on page 62):

a Pick up to 11 harvested samples at random from both sections of the HT IFC (see Figure 4 on page 30) to run on the Agilent Bioanalyzer.

NOTE (Optional) If performing a tube control (see Appendix A on page 51), make sure to reserve 2 sample wells on the chip to run each tube control reaction.

b Measure the cDNA concentration of each sample on the chip, estimated with a size range of ≈200–9,000 bp.

c Calculate the average yield of each measured sample. Use this average value as the effective concentration for all 20 harvested samples.

2 Dilute 2 µL of the 20 harvested samples to 0.1–0.2 ng/µL in the same volume of C1 Harvest Reagent, based on the effective concentration calculated in step 1c.

As an example, if your average yield from the harvested samples is 1.5 ng/µL, then you can use a 10-fold dilution to fall within the desired size range. In this example, you can dilute 2 µL of each sample in 18 µL of C1 Harvest Reagent.

IMPORTANT To ensure optimal results, a minimum volume of 2 µL of harvested sample is required.

NOTE Keep diluted libraries on ice until ready to use, or store at –20 °C long term.

Perform Tagmentation

IMPORTANT To ensure reliable results, follow the Best Practices on page 18.

NOTE If you are running the optional tube controls (see Appendix A on page 51), prepare enough extra volume in the following procedures (plus 25% overage).

Prepare cDNA for Tagmentation

IMPORTANT Warm Tagment DNA Buffer and Neutralize Tagment (NT) Buffer to room temperature. Visually inspect NT Buffer to ensure there is no precipitate. If there is precipitate, vortex until all particulates are resuspended.

1 After thawing, ensure all reagents are adequately mixed by gently inverting the tubes 3–5 times, followed by centrifuging the tubes briefly to collect the contents.

2 Label a new 96-well PCR plate “Library Prep”.

3 In a 1.5 mL microtube, combine the components of the tagmentation pre-mix. You will need enough pre-mix for each sample you wish to sequence, plus 25% overage. For 20 samples (entire HT IFC):

Reagent	Volume per Sample (µL)	Volume for 20 Samples (plus 25% overage; µL)
PRE-MIX		
Tagment DNA Buffer	2.5	62.5
Amplification Tagment Mix	1.25	31.25
Diluted Sample	1.25	—
**Total**	**5.0**	**—**

4 Vortex at low speed for 20 seconds and centrifuge the tube to collect contents.

5 Pipet 3.75 µL of the pre-mix into each of the 20 wells of the Library Prep plate (for the 20 samples), using a fresh pipette tip for each well.

NOTE We recommend that you maintain the same 20-well layout for tracking purposes (see Figure 8 on page 39 and Figure 9 on page 46). If you customize the Library Prep plate layout, track the positions carefully.

6 Pipet 1.25 µL of the diluted sample from the diluted sample plate to the Library Prep plate.

7 Seal plate and vortex at medium speed for 20 seconds. Centrifuge at ≈ 3,000 x *g* for 5 minutes to remove bubbles.

8 Place the Library Prep plate in a thermal cycler and run the following program:

Temperature	Time
55 °C	10 min
10 °C	Hold

NOTE Ensure that the thermal cycler lid is heated during the incubation.

9 Aliquot equal volumes of NT Buffer into each tube of an 8-tube strip, using a fresh pipette tip for each well. You will need 1.25 µL of NT Buffer for each sample you wish to sequence, plus 25% overage. For 20 samples (entire HT IFC):

Reagent	Volume per Sample (µL)	Volume per 20 Samples (plus 25% overage; µL)
Library Prep plate	5.0	—
NT Buffer	1.25	31.25
**Total**	**6.25**	**—**

10 Once the sample reaches 10 °C, immediately pipet 1.25 µL of the NT Buffer to each of the tagged and fragmented (tagmented) samples to quickly neutralize the samples.

11 Seal plate and vortex at medium speed for 20 seconds. Centrifuge at ≈ 3,000 x *g* for 5 minutes.

Amplify the Tagmented cDNA

IMPORTANT For Index Primer selection criteria and handling instructions, carefully read the Nextera XT DNA Library Preparation Guide before proceeding to PCR amplification of the tagmented cDNA.

NOTE We recommend that you maintain the same 20-well layout for tracking purposes (see also Figure 8 on page 39). If you customize the Library Prep plate layout and index usage, track the positions and indices carefully.

1 Pipet 3.75 µL of Nextera PCR Master Mix (NPM) into each sample well of the Library Prep plate. For 20 samples (entire HT IFC):

Reagent	Volume per Sample (µL)	Volume per 20 Samples (plus 25% overage; µL)
Library Prep plate	6.25	—
NPM	3.75	93.75
**Total**	**10.0**	**—**

NOTE You can aliquot equal volumes of NPM into each tube of an 8-tube strip for ease of pipetting. You will need 3.75 µL of NPM for each tagmented sample you wish to sequence, plus 25% overage.

2 Add primers to the Library Prep plate (see Figure 9):

a Pipet 1.25 µL of Enrichment Primer (EP) to each of the 20 sample libraries on the Library Prep plate.

b Pipet 1.25 µL of the appropriate Index Primer from Set A or Set B to each of the 20 sample libraries on the Library Prep plate. Only 20 of the 24 total indices are required. We recommend using the following indices:

- **Set A:** N701–N707, N710–N712, and N714–N715
- **Set B:** N716, and N718–N724

NOTE (Optional) If performing a tube control (see Appendix A on page 51), randomly select two of the unused indices from Set B (N726–N729) and pipet 1.25 µL of the appropriate Index Primer to each of the recommended wells. Make sure to track the positions and indices carefully.

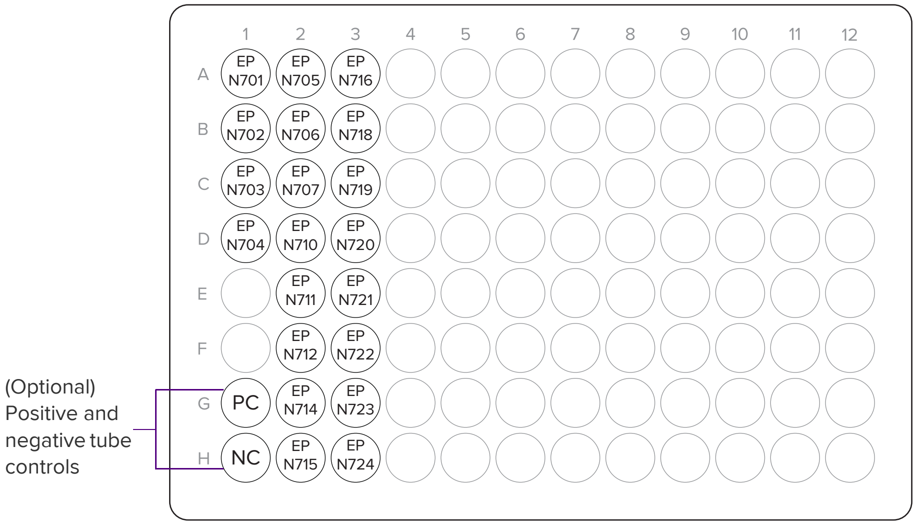

Figure 9. Index Primer pipette map and index assignment

3 Seal the plate with adhesive film and vortex at medium speed for 20 seconds. Centrifuge at ≈ 3,000 x *g* for 2 minutes.

4 Place the plate into a thermal cycler and perform PCR amplification:

Temperature	Time	Cycles
72 °C	3 min	1
95 °C	30 sec	1
95 °C	10 sec	12
55 °C	30 sec	
72 °C	60 sec	
72 °C	5 min	1
10 °C	Hold	—

NOTE Ensure that the thermal cycler lid is heated during the incubation.

STOPPING POINT Amplified products can be stored long-term at −20 °C.

Pool and Clean Up the Library

IMPORTANT To ensure reliable results, follow the Best Practices on page 18.

NOTE If you are running the optional tube controls (see Appendix A on page 51), prepare enough extra volume in the following procedures (plus 25% overage).

Pool and Clean Up

1 Determine number of samples to be pooled based on desired sequencing depth and sequencer throughput.

NOTE Keep in mind that each sample from the HT IFC will include up to 40 cells.

2 Warm AMPure XP beads up to room temperature and vortex for 1 minute.

3 In a 1.5 mL microtube, make the library pool as shown below:

a Pipet the appropriate volume from each sample (column 2) according to the number of samples to be pooled (column 1).

b Add the required amount of AMPure XP beads (column 4) to the pooled library.

1. Number of Samples to be Pooled	2. Volume per Sample (µL)	3. Total Library Pool Volume (µL)	4. AMPure Bead Volume for Cleanup (≈ 90% of total library pool volume; µL)
8	4	32	29
12	4	48	44
16	2	32	29
20 (entire HT IFC)	2	40	36

4 Mix well by pipetting up and down 5 times, then incubate the bead mix at room temperature for 5 minutes.

5 Place the tube on a magnetic stand for 2 minutes, then carefully remove the supernatant without disturbing the beads.

6 Add 180 µL of freshly prepared 70% ethanol and incubate for 30 seconds on the magnetic stand, then remove the ethanol.

7 Repeat step 6.

8 Allow the beads to air-dry on bench for 10–15 minutes.

9 Elute the samples by adding the required volume of DNA Dilution Reagent according to the number of samples pooled:

Number of Samples Pooled	DNA Dilution Reagent Volume for Elution (= original total library pool volume; µL)
8	32
12	48
16	32
20 (entire HT IFC)	40

10 Vortex the tube for 3 seconds and incubate it for 2 minutes at room temperature.

11 Plate the tube on a magnetic stand for 2 minutes, then transfer the entire volume of supernatant to another microtube.

Repeat Cleanup

1 Add the required volume of AMPure XP beads according to the number of samples pooled:

Number of Samples Pooled	AMPure Bead Volume for Cleanup (≈ 90% of elution volume; µL)
8	29
12	44
16	29
20 (entire HT IFC)	36

2 Mix well by pipetting up and down 5 times, then incubate the bead mix for 5 minutes at room temperature.

3 Place the tube on a magnetic stand for 2 minutes, then carefully remove the supernatant without disturbing the beads.

4 Add 180 µL of freshly prepared 70% ethanol and incubate for 30 seconds on the magnetic stand, then remove the ethanol.

5 Repeat step 4.

NOTE Some beads may be lost during ethanol cleanup.

6 Allow beads to air-dry on bench for 10–15 minutes.

7 Elute the samples by adding the required volume of DNA Dilution Reagent according to the number of samples pooled:

Number of Samples Pooled	Volume of DNA Dilution Reagent for Elution (≈ 1.5x original pool volume; µL)
8	48
12	72
16	48
20 (entire HT IFC)	60

8 Remove the tube from the magnetic stand and vortex the tube for 3 seconds, then incubate at room temperature for 2 minutes.

9 Place the tube on the magnetic stand for 2 minutes, then carefully transfer the supernatant to another microtube labeled "SC Lib."

10 Run an Agilent High Sensitivity DNA Chip on undiluted samples in triplicate to check for library size distribution and quantity (see Figure 11 on page 62). See the Agilent 2100 Bioanalyzer 2100 Expert User's Guide for this step.

Illumina Sequencing Requirements and Recommendations

- See the Illumina Nextera XT DNA Library Preparation Guide to determine the appropriate library concentration for sequencing.
- For the C1 mRNA Sequencing High Throughput Demultiplexer API to function correctly (see Appendix B on page 55), each sequencing sample name in the Illumina sample sheet must begin with "COL<N>_<sample name>," where:
    - **COL<N>** = column number from the HT IFC (0-padded to 2 digits)
    - **<sample name>** = your sample name

  For example, if you are interested in sequencing Column 1 harvested from the HT IFC for sample S1, you will enter "COL01_S1" in the sample sheet in order to generate a FASTQ file starting with the required information.
- We recommend that you run the following number of cycles for each of the Illumina MiSeq or HiSeq sequencer reads (R1 and R2, see Figure 2 on page 11):
    - **R1 reads:** 25–30 cycles, to generate sufficient length to identify the sample based on the row barcode sequence.
    - **R2 reads:** 75–100 cycles, to generate sufficient length to accurately map the cDNA fragment (average cDNA insertion size is ≈ 80 bp).

Appendix A: (Optional) Run the Tube Controls

Introduction

The tube controls are used as positive and negative controls for the C1 Single-Cell mRNA Seq HT reagents and workflow performed off the HT IFC (see page 12). The results from the multi-cellular positive tube control can also be compared with the single-cell samples harvested from the IFC. You prepare two tube controls: one with cells (tube 1; positive control or PC), and one without cells (tube 2; no template control or NTC). You perform the tube control reactions (lysis, RT, and preamplification) off the IFC using the same chemistry you use to process the single cells on the IFC.

Wash the Cell Mix

We recommend that you wash the cell mix you prepared with Suspension Reagent and reserved after loading the HT IFC (see step 5 on page 33) before use in the tube control reactions, using 1X PBS or similar wash buffer appropriate for your cell type:

1. Pellet the reserved cell mix. Speeds and durations may vary. We suggest centrifuging the 1.5 mL microtube containing the cells at 300 x *g* for 5 minutes.
2. Gently remove the supernatant without disturbing the cell pellet.
3. Resuspend cells in 1 mL of wash buffer by gently pipetting up and down at least 5 times. This is wash 1.
4. Repeat steps 1–2.
5. Resuspend cells again in 1 mL of wash buffer by gently pipetting up and down at least 5 times. This is wash 2.
6. Repeat steps 1–2.
7. Resuspend cells in wash buffer to approximately 50% of original volume to achieve a concentration of 250–500 cells/µL.

   NOTE Cells may be counted by any preferred method. If an established cell counting protocol does not exist, we suggest using the disposable hemocytometer C-Chip by INCYTO. See incyto.com/product/product02_detail.php for instructions for use.

8. Keep the washed cell mix on ice until use.

Perform the Tube Control Reactions

1 Prepare two tube controls by combining lysis reagents and thermal cycling them:

a Prepare cell lysis mix:

Components	Tube 1: PC (positive control; µL)	Tube 2: NTC (no template control; µL)
Washed cell mix (see page 51)	2.1	—
Cell Rinsing Reagent ○	—	2.1
Lysis Mix A plus diluted barcodes (for the tube control, see page 24)	3.6	3.6
**Total**	**5.7**	**5.7**

b Mix gently and centrifuge briefly to collect contents.

c In a thermal cycler, run the following cell lysis protocol:

Temperature	Time
72 °C	3 min
4 °C	10 min
25 °C	1 min
4 °C	Hold

2 Combine RT Mix B with cell lysis products from step 1c:

a Prepare RT reaction:

Components	Tube 1: PC (µL)	Tube 2: NTC (µL)
Cell lysis products (from step 1c)	5.7	5.7
RT Mix (Mix B, see page 25)	6.4	6.4
**Total**	**12.1**	**12.1**

b Mix gently and centrifuge briefly to collect contents.

c In a PCR thermal cycler, run the following RT protocol:

Temperature	Time
42 °C	90 min
4 °C	Hold

STOPPING POINT If you program to harvest the next day (see page 36), you can store the RT reaction overnight at 4 °C until you are ready to run the PCR reaction.

3 After the thermal cycle protocol has finished, combine the following PCR reaction components in two tubes of an unused 8-tube strip:

Components	Tube 1: PC (µL)	Tube 2: NTC (µL)
RT reaction (from step 2c)	6.0	6.0
Preamplification Mix I (Mix D, see page 26)	7.25	7.25
**Total**	**13.25**	**13.25**

a Mix gently and centrifuge briefly to collect contents, then run the following PCR protocol:

Temperature	Time	Cycles
98 °C	1 min	1
98 °C	20 sec	
59 °C	4 min	4
68 °C	6 min	
72 °C	10 min	1
4 °C	Hold	1

b Centrifuge briefly to collect contents.

Dilute the Products and Process the Tube Controls

1 Transfer prepared material (from step 3b above) to a post-PCR room.

2 Vortex the prepared PCR products for 3 seconds and centrifuge briefly to collect contents.

3 Combine the following reagents to dilute the PCR products:

Components	Tube 1: PC (µL)	Tube 2: NTC (µL)
PCR product (from step 1)	1.0	1.0
Cleanup Mix (Mix C, see page 26)	4.5	4.5
**Total**	**5.5**	**5.5**

4 Mix gently and centrifuge briefly to collect contents.

5 Transfer the diluted tube control samples to the appropriate wells of the harvest plate (see Harvest, Clean up, and Preamplification off the C1 System on page 37), then continue with the protocol and process the tube control samples along with the harvested samples.

Appendix B: Data Analysis

Analysis Workflow

The workflow for analysis of RNA sequencing data from the C1 mRNA Seq HT IFC protocol is as follows:

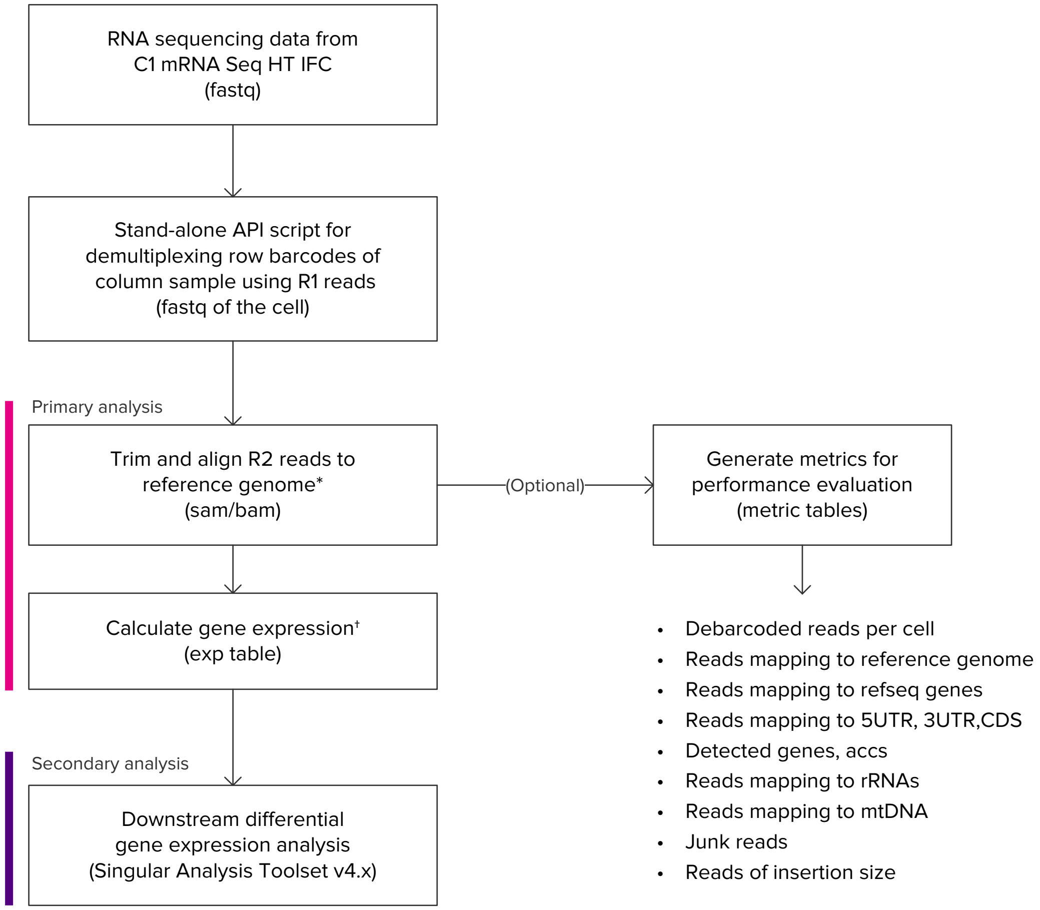

Primary analysis recommendations:
* Alignment by Tophat/rsem
† Gene expression calculation by Cufflink/rsem

Stand-alone API Script for Demultiplexing

Initially, only 20 samples are demultiplexed from the Illumina MiSeq or HiSeq sequencer reads (R1 and R2) because the sequencer only demultiplexes Nextera indices (columns). The C1 mRNA Sequencing High Throughput Demultiplexer Perl script API available for download from Script Hub™ demultiplexes the individual single-cell samples from each column using the cell barcodes on the R1 reads. The script allows you to separate the large FASTQ file generated from each single-column harvest into 40 separate FASTQ files, one for each row in the HT IFC.

Download the Script:

Go to fluidigm.com/c1openapp/scripthub and navigate to the C1 mRNA Sequencing High Throughput Demultiplexer script. You must be logged in to your Fluidigm account to download a script.

Filename of Script:

"`mRNASeqHT_demultiplex.pl`"

Description:

The script is used to demultiplex raw FASTQ files into multiple FASTQ files, named by COL<N>_ROW<N>_SAMPLENAME (see page 50).

Use Case Synopsis:

```
perl mRNASeqHT_demultiplex.pl –i [INPUT_DIR] –o [OUTPUT_DIR]

[required]

-i              input dir of fastq data
-o              output dir of demultiplexed fastq data
```

Additional Analysis Recommendations

- Before aligning R2 reads to the reference genome, trim the polyA stretch from the 3′ end of the R2 reads (average cDNA insertion size is ≈ 80 bp).
- Use only R2 reads for the downstream analysis for end-counting in transcriptome expression.
- Use the demultiplexed R2 reads for expression analysis, as described in the analysis workflow (see page 55).

For more information on downstream differential gene expression analysis, see the Singular Analysis Toolset User Guide (version 4.x or later).

Appendix C: HT IFC Pipetting Maps

Priming Map

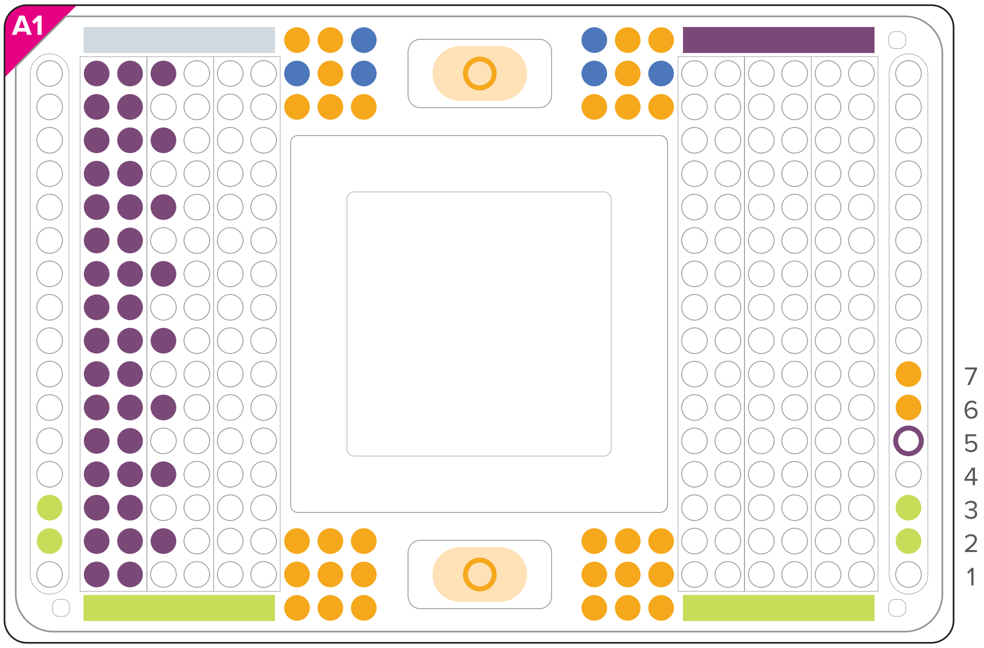

- Valve Fluid, 200 µL
- Valve Fluid, 20 µL
- Stability Solution, 20 µL
- Preloading Reagent, 5 µL
- Preloading Reagent, 20 µL
- C1 Harvest Reagent, 180 µL
- Preloading Reagent, 150 µL
- 1X Blocking Reagent, 180 µL
- 1X Blocking Reagent, 20 µL

Loading Map

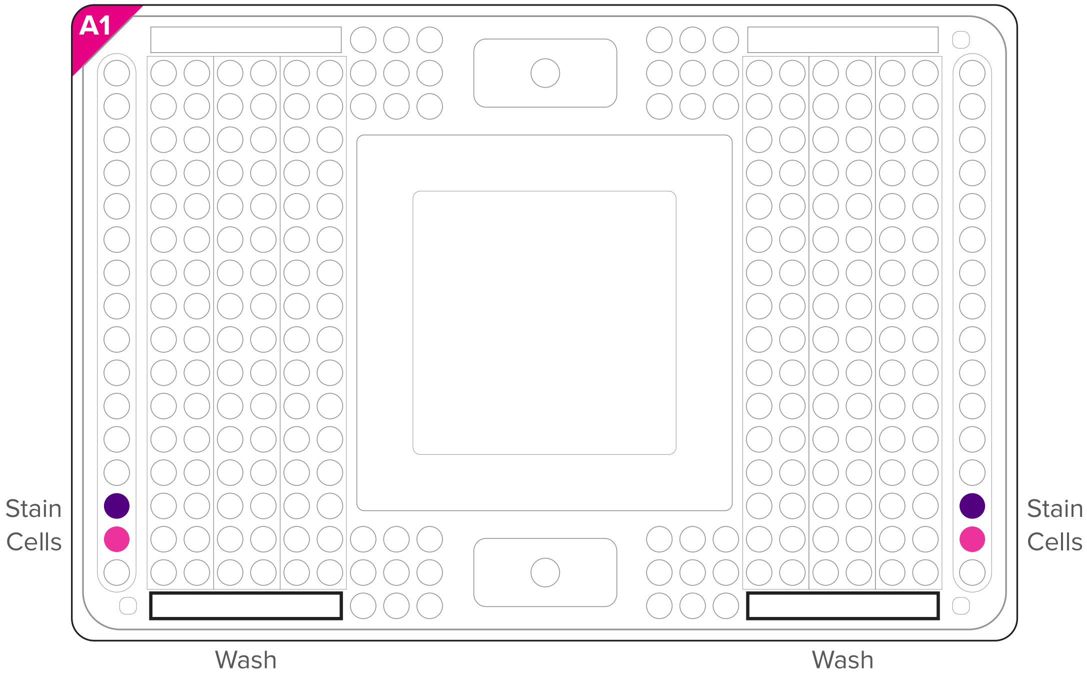

- Cell Rinsing Reagent, 180 µL
- (Optional) LIVE/DEAD Staining Solution, 20 µL
- Cell Mix(es), 20 µL

Lysis, RT, and Cleanup Map

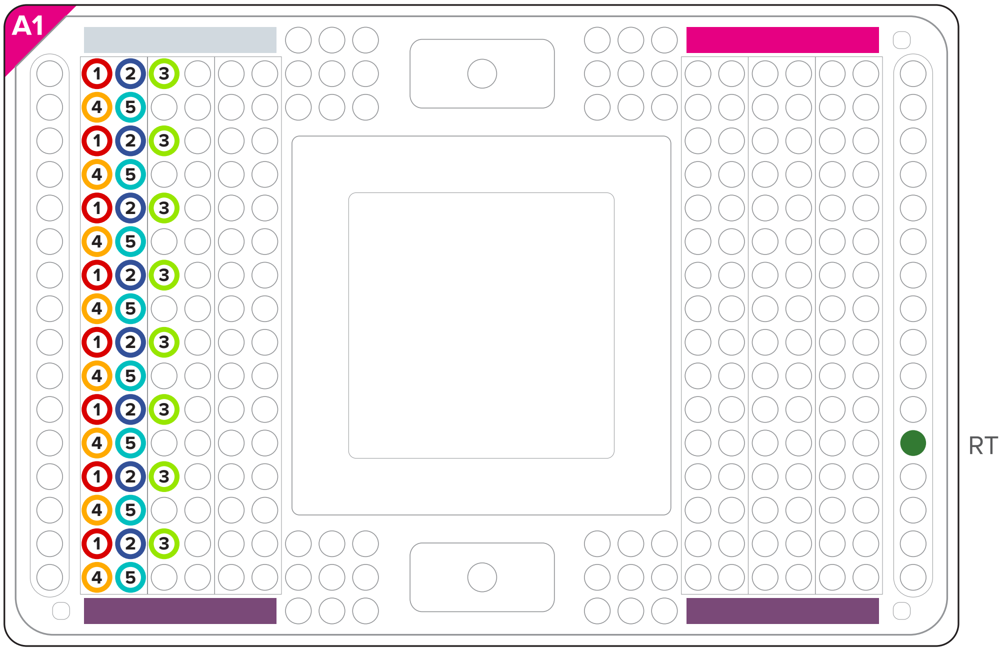

① ② ③ ④ ⑤ Lysis Mix A Plus Diluted Barcodes, 4.2 µL

RT Mix B, 20 µL

Preamplification Mix I (Mix D), 130 µL

Preloading Reagent, 150 µL

Cleanup Mix C, 180 µL

Harvesting Map

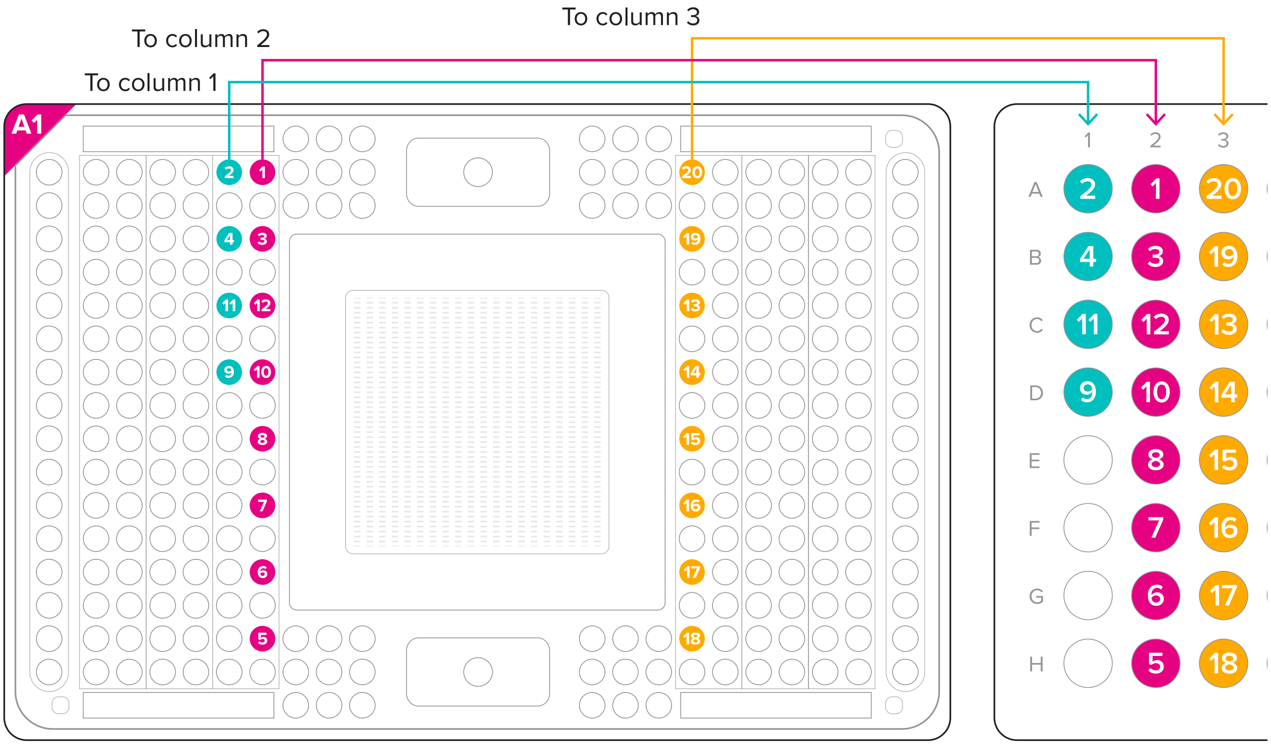

Column Attribution Map

	1	2	3	4	5	6	7	8	9	10	11	12
A	2	1	20									
B	4	3	19									
C	11	12	13									
D	9	10	14									
E		8	15									
F		7	16									
G	PC	6	17									
H	NC	5	18									

(Optional) Positive and negative tube controls

Index Primer and Assignment Map

	1	2	3	4	5	6	7	8	9	10	11	12
A	EP N701	EP N705	EP N716									
B	EP N702	EP N706	EP N718									
C	EP N703	EP N707	EP N719									
D	EP N704	EP N710	EP N720									
E		EP N711	EP N721									
F		EP N712	EP N722									
G	PC	EP N714	EP N723									
H	NC	EP N715	EP N724									

(Optional) Positive and negative tube controls

Appendix D: Library Prep Examples

Typical Agilent Bioanalyzer Traces

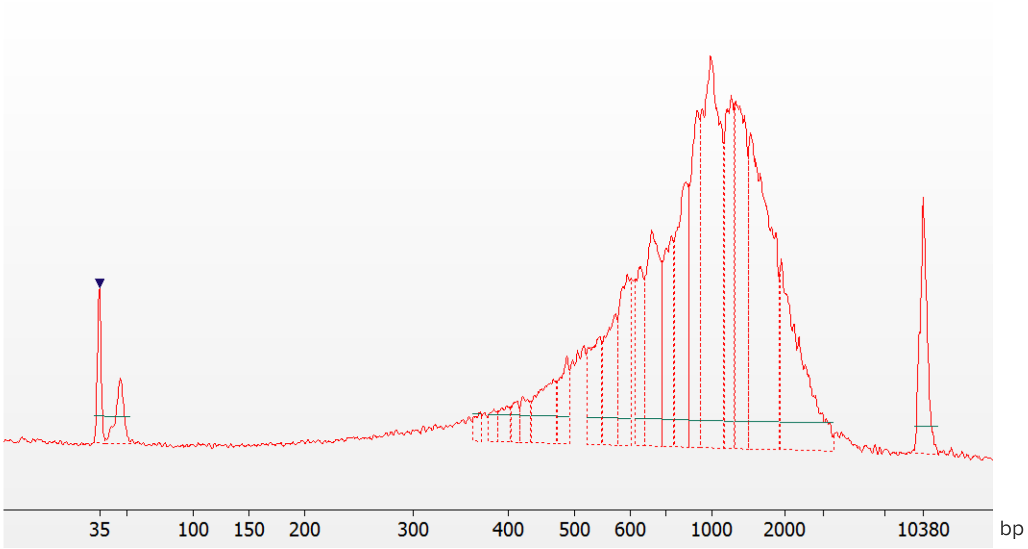

Figure 10. A typical cDNA harvested from HT IFC (after preamplification off the HT IFC)

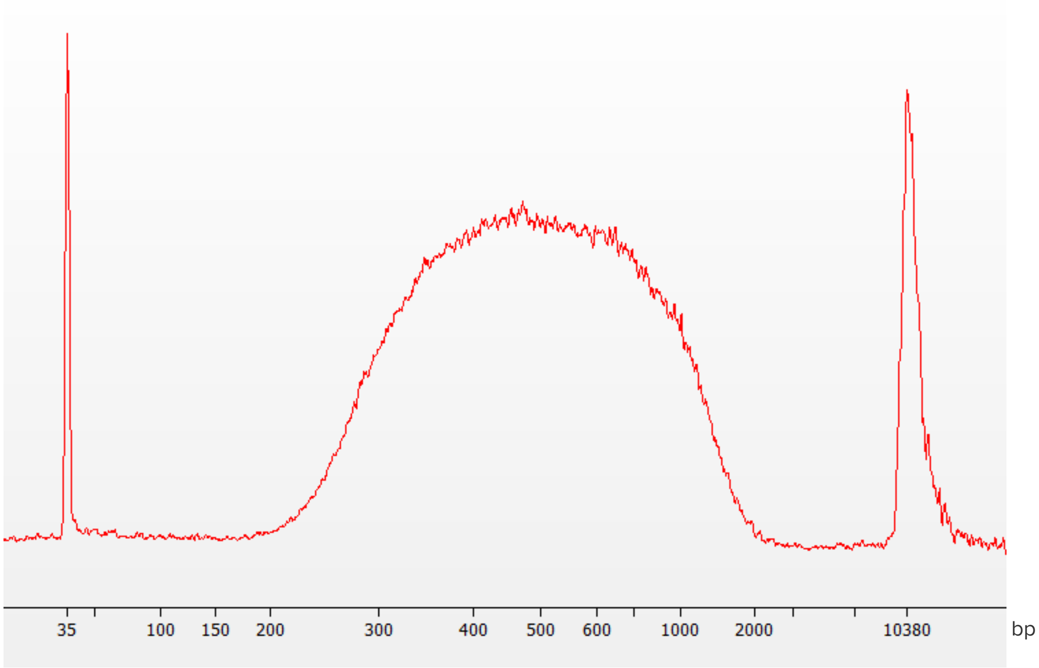

Figure 11. Typical library size distribution (after purification)

Appendix E: C1 Single-Cell mRNA Seq HT Reagent Kit PN 101-0063

NOTE

- The C1 Single-Cell mRNA Seq HT Reagent Kit is shipped in five boxes: Module 1, Module 2, Module 3, Module 4, and Module 5.
- When ordering the modules from Fluidigm, use the kit part number: 101-0063.
- The modules in this kit provide the necessary reagents to run mRNA Seq HT chemistry on five C1 HT IFCs.
- For storage conditions, see Required Reagents on page 15. For kit usage, see page 19.

Module 1 (PN 101-0059)

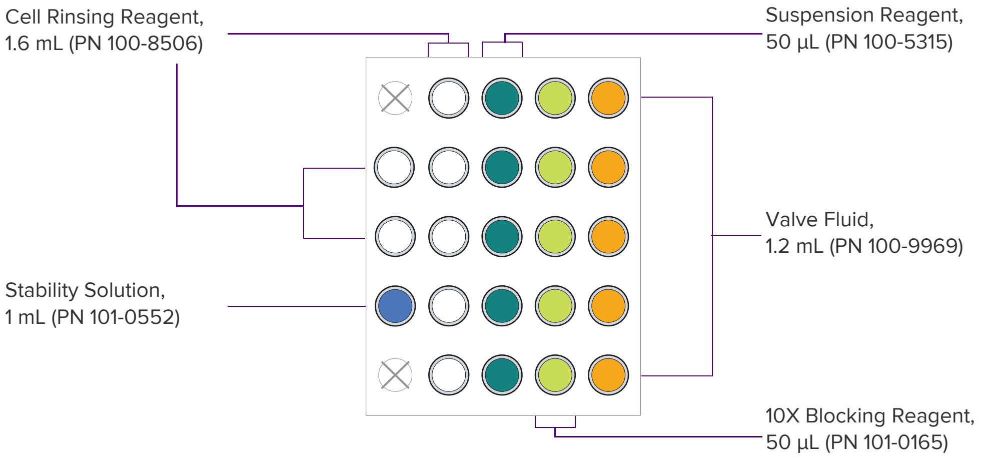

Module 2 (PN 101-0060)

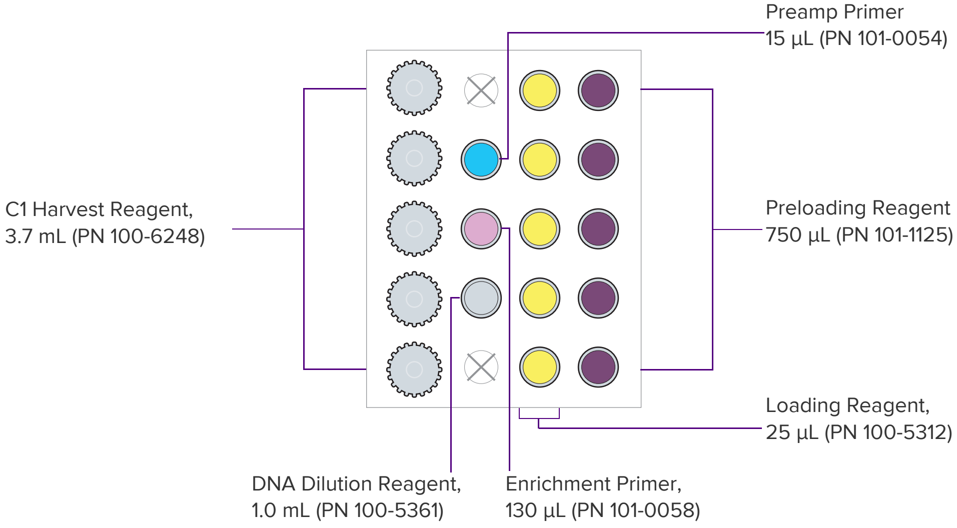

Appendix F: HT IFC Types and Related Scripts

There is currently one C1 system-compatible HT IFC for medium single cells:

Cell Size/IFC Name and Part Numbers	Barcode (prefix)	Script Names	Description
**For mRNA Seq HT protocol:**			
**Medium** (10–17 µm) C1 IFC for mRNA Seq HT (10–17 µm) PN 101-0221	1910x	mRNA Seq HT: Prime (1910x)	Priming the control line and cell capture channels of the 10–17 µm mRNA Seq HT IFC (1910x)
		mRNA Seq HT: Cell Load (1910x)	Cell loading and washing without staining for PCR of 10–17 µm diameter cells (1910x)
		mRNA Seq HT: Cell Load & Stain (1910x)	Cell loading, staining, and washing for mRNA sequencing of 10–17 µm diameter cells (1910x)
		mRNA Seq HT: RT & Amp (1910x)	Loading, thermal, and harvest protocol for single-cell lysis, reverse transcription, and cDNA amplification for mRNA sequencing of 10–17 µm cells (1910x)

Appendix G: Related Documentation

cDNA Synthesis

- Agilent 2100 Bioanalyzer 2100 Expert User's Guide (Agilent Technologies PN G2946-90004)
- C1 System User Guide (Fluidigm PN 100-4977)
- C1 mRNA Sequencing DataSheet (Fluidigm PN 101-0984)
- ArrayControl Spots and Spikes (Thermo Fisher Scientific PN AM1781)
- Fluidigm Single-Cell Preparation Guide (Fluidigm PN 100-7697)
- INCYTO Disposable Hemocytometer, incyto.com/product/product02_detail.php
- LIVE/DEAD Viability/Cytotoxicity Kit, for mammalian cells (Thermo Fisher Scientific PN L-3224)
- Minimum Specifications for Imaging Cells in Fluidigm Integrated Fluidic Circuits Specification Sheet (Fluidigm PN 100-5004)
- Singular Analysis Toolset User Guide (Fluidigm PN 100-5066)
- Using a Microscope with an Automated Stage Quick Reference (Fluidigm PN 100-6130)

DNA Sequencing

- Illumina Nextera XT DNA Library Preparation Guide (Illumina PN 15031942)

Appendix H: Safety

General Safety

In addition to your site-specific safety requirements, Fluidigm recommends the following general safety guidelines in all laboratory and manufacturing areas:

- Use personal protective equipment (PPE): safety glasses, fully enclosed shoes, lab coats, and gloves.
- Know the locations of all safety equipment (fire extinguishers, spill kits, eyewashes/showers, first-aid kits, safety data sheets, etc.), emergency exit locations, and emergency/injury reporting procedures.
- Do not eat, drink, or smoke in lab areas.
- Maintain clean work areas.
- Wash hands before leaving the lab.

Instrument Safety

WARNING Do not modify this device. Unauthorized modifications may create a safety hazard.

CAUTION HOT SURFACE. The C1 thermal cycler chuck gets hot and can burn your skin. Use caution when working near the chuck.

CAUTION PINCH HAZARD. The C1 door and shuttle can pinch your hand. Make sure your fingers, hand, shirtsleeve, etc., are clear of the door and shuttle when loading or ejecting a IFC.

WARNING BIOHAZARD. If you are putting biohazardous material on the instrument, use appropriate personal protective equipment and adhere to *Biosafety in Microbiological and Biomedical Laboratories* (BMBL) from the Centers for Disease Control and Prevention and to your lab's safety protocol to limit biohazard risks. If biohazardous materials are used, properly label the equipment as a biohazard. For more information, see the BMBL guidelines at: cdc.gov/biosafety/publications/index.htm

For a full list of the symbols on the instrument, refer to the C1 System User Guide (PN 100-4977).

Chemical Safety

Read and comprehend all safety data sheets (SDSs) by chemical manufacturers before you use, store, or handle any chemicals or hazardous materials.

Wear personal protective equipment (gloves, safety glasses, fully enclosed shoes, lab coats) when handling chemicals.

Do not inhale fumes from chemicals. Use adequate ventilation, and return caps to bottles immediately after use.

Check regularly for chemical spills or leaks. Follow SDS recommendations for cleaning up spills or leaks.

Disposal of Products

Used IFCs should be handled and disposed of in accordance with federal, state, regional, and local laws for hazardous waste management and disposal.

Do not dispose of this product in unsorted municipal waste. This equipment may contain hazardous substances that could affect health and the environment. Use appropriate take-back systems when disposing of materials and equipment.

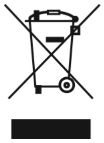

Learn more at fluidigm.com/compliance

For technical support visit fluidigm.com/support
